# Two-dimensional chiral topological superconductivity in Shiba lattices

**DOI:** 10.1038/ncomms12297

**Published:** 2016-07-28

**Authors:** Jian Li, Titus Neupert, Zhijun Wang, A. H. MacDonald, A. Yazdani, B. Andrei Bernevig

**Affiliations:** 1 Department of Physics, Princeton University, Princeton, New Jersey 08544, USA; 2 Princeton Center for Theoretical Science, Princeton University, Princeton, New Jersey 08544, USA; 3 Department of Physics, University of Zurich, Winterthurerstrasse 190, CH-8057 Zurich, Switzerland; 4 Department of Physics, University of Texas at Austin, Austin, Texas 78712, USA

## Abstract

The chiral p-wave superconductor is the archetypal example of a state of matter that supports non-Abelian anyons, a highly desired type of exotic quasiparticle. With this, it is foundational for the distant goal of building a topological quantum computer. While some candidate materials for bulk chiral superconductors exist, they are subject of an ongoing debate about their actual paring state. Here we propose an alternative route to chiral superconductivity, consisting of the surface of an ordinary superconductor decorated with a two-dimensional lattice of magnetic impurities. We furthermore identify a promising experimental platform to realize this proposal.

## Abstract (web summary)

Chiral p-wave superconductors may form the basis for topological quantum computers however one has yet to be identified. Here, the authors propose that such a state may be created by a two-dimensional array of magnetic adatoms on the surface of a superconductor with strong spin-orbit coupling.

## Graphical abstract

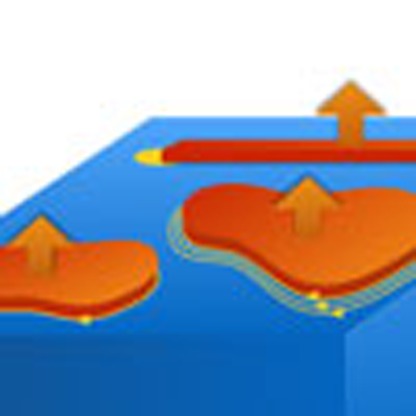

The chiral p-wave superconductor in two dimensions (2D) and the closely related fractional quantum Hall Pfaffian state at filling fraction *ν*=5/2 are the archetypal examples of topologically ordered states of matter that support non-Abelian anyonic excitations^1^,^2^,^3^. The theoretical exploration of these states has shaped our understanding of topological order and is foundational for the distant goal of building a topological quantum computer^4^,^5^,^6^. In contrast to the *ν*=5/2 fractional quantum Hall state, however, to date chiral *p*-wave superconductivity has not been confirmed in any experimental system. The most prominent candidate system, superconducting Sr_2_RuO_4_ (ref. 7), has been the subject of an ongoing debate about its actual paring state^8^,^9^,^10^,^11^.

Chiral superconductors break time-reversal symmetry. This hinders the formation of Cooper pairs, since orbital (and possibly paramagnetic) pair-breaking effects can come into play. Depairing is also the main hurdle for realizing a line of proposals in which layered heterostructures involving ferromagnets and *s*-wave superconductors are used to build an artificial 2D *p*-wave superconductor^12^,^13^,^14^,^15^. The guiding principle for these proposals is to design a band structure with a single normal-state Fermi surface with no spin-degeneracy. If Rashba spin-orbit coupling is present so that the states on this Fermi surface are not fully spin-polarized, and if *s*-wave superconductivity is proximity-induced in such a system, the effective pairing near the single Fermi surface is equivalent to that of a chiral *p*-wave superconductor.

In one dimension (1D), based on the principle of combining spin-orbit coupling and externally applied magnetic fields, various groups have proposed engineering artificial realizations of *p*-wave superconductors^16^,^17^,^18^,^19^,^20^,^21^,^22^. An experimental realization of these proposals employing semiconductor nanowires with strong spin-orbit coupling^23^ has reported Majorana fermion signatures. In this setup, the externally applied magnetic field has to be rather small (to avoid suppressing superconductivity). As the phase-space for the existence of a topological superconductor is controlled by the Zeeman gap^24^, these systems require a delicate balance of the parameters involved (spin-orbit coupling, magnetic field and chemical potential) in order to create the topological superconductor.

Recently, a 1D topological superconductor was realized in a system that is quite distinct but employs similar microscopic ingredients—spin-orbit coupling, ferromagnetism and s-wave superconductivity^25^. A chain of magnetic Fe atoms is deposited on the surface of an *s*-wave superconductor with strong spin–orbit interactions. The Fe chain is ferromagnetically ordered^25^ with a large magnetic moment, which takes the role of the magnetic field in the nanowire experiments. Unlike previous proposals, this magnetic field is mostly localized on the Fe chain, with small leakage outside. Superconductivity is not destroyed along the chain. In this setup, the energy scale of the exchange coupling of the Fe atoms is typically much larger than that of the Rashba spin-orbit coupling and the superconducting pairing. The ferromagnetically ordered Fe atoms induce localized Shiba states within the gap of the superconductor^26^,^27^,^28^. The hybridization of these states forms the band structure of a 1D *p*-wave superconductor that supports Majorana end states^29^,^30^. Because the Fe bands are fully spin split, no additional control over the chemical potential is necessary. A similar scenario applies when the Fe orbitals are magnetic but itinerant^24^.

In this article, we point out that this strategy can also be successful in 2D. Magnetic adatoms on the surface of a superconductor with strong spin-orbit coupling, when arranged in a 2D lattice, can yield a 2D topological chiral *p*-wave superconductor whose chiral Majorana edge modes can be observed in scanning tunneling microscope measurements. To shed light on the rich range of possibilities, we analyse the topological properties of a system with dense local moments that are exchange coupled to a model 2D superconductor, demonstrating that topological superconductors with higher Chern numbers, and consequently multiple chiral Majorana edge channels, can easily occur. We are also able to analyse the model's dilute magnetic impurity limit analytically and obtain numerical topological phase diagrams for intermediate impurity concentrations. Based on density-functional-theory (DFT) calculations, we further propose realizing 2D topological chiral p-wave superconductors experimentally by depositing transition metal adatoms on superconducting Pb. The type of magnetic ion can be varied to access different strengths of the magnetic moment. In the case of Fe adatoms on a Pb (111) surface, we show that strong magnetic order in general leads to an odd number of 2D Fermi surface segments. As a consequence the proximity-induced superconducting phases can have nonzero Chern numbers and chiral Majorana edge modes.

## Results

### Model Hamiltonian

**Figure 1:**
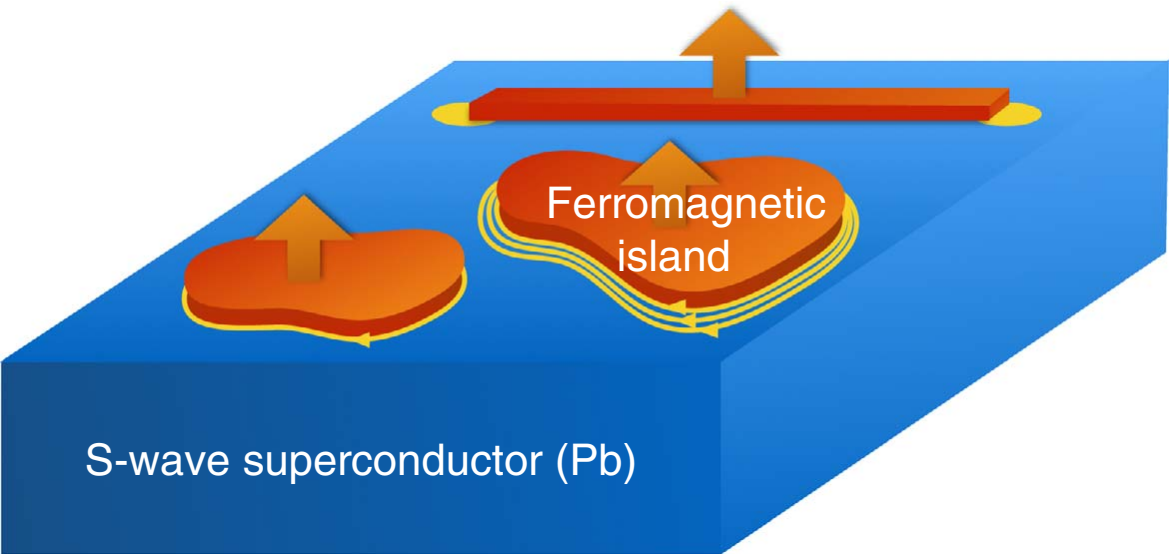
Topological superconductivity in Shiba lattice systems. A very thin layer of magnetic adatoms, such as Fe or Co, is deposited on the surface of a conventional *s*-wave superconductor with strong spin-orbit coupling, such as Pb, and forms a one-dimensional chain or a two-dimensional island that is ferromagnetically ordered (represented by the upward arrows). The resultant lattice of magnetic-impurity-induced bound states, or Shiba lattice, generically bears chiral topological superconductivity and the associated chiral Majorana boundary modes (represented by yellow dots or lines with unidirectional arrows). In two dimensions, one or many Majorana modes may appear depending on the density of the magnetic adatoms. These modes can be detected by tunneling techniques.

We first present a model system that bears a number of generic features of superconductor surfaces with ferromagnetically ordered magnetic adatoms (see Fig. 1). To render the analytical calculations tractable, we consider a Hamiltonian that models only the surface layer of a bulk 3D *s*-wave superconductor on which the *s*-wave superconducting order parameter Δ is induced from the bulk. On this superconducting layer, we model the magnetic impurities as classical spins whose only interaction with the electrons in the superconductor is through Zeeman-like couplings^26^,^27^,^28^. Employing a tight-binding description on a 2D square lattice Λ that is spanned by the primitive lattice vectors 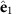 and 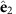, we consider the mean-field Hamiltonian


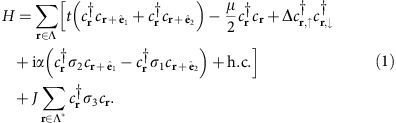


Here, 
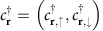
 is a spinor of the creation operators for electrons at site **r** with spin ↑, ↓, and *σ*_*i*_,*i*=1,2,3, are the three Pauli matrices. We denote by *t* the nearest neighbor hopping integral in the superconductor, *μ* the chemical potential, and *α* the strength of the Rashba spin-orbit coupling. Classical magnetic moments ferromagnetically aligned normal to the plane in the *σ*_3_ direction are positioned on a sublattice Λ* of Λ and are exchange-coupled to electrons via the term proportional to *J*. Drawing experience from the 1D situation and the *ab-initio* calculations presented below, the physically relevant hierarchy of energy scales that we consider here is given by


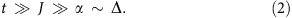


### Dense impurity limit

As a warmup, it is instructive to consider the simplest situation in which each lattice site is coupled to a magnetic moment, i.e., Λ=Λ*. This limit is representative of system with self-assembled islands of magnetic adatoms. Its consideration allows us to highlight the difference between the regime in equation (2), and that of small *J* that has been studied previously^12^,^14^,^15^. In particular, it is possible to access a phase with Chern number 2 in the large *J* limit. For the case Λ=Λ* and *α*=0, the Hamiltonian in equation (1) is gapless at zero energy with nodal lines in momentum space defined by (see Supplementary Note 1)


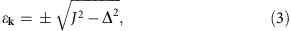


where *ɛ*_**k**_=2*t*(cos *k*_1_+cos *k*_2_)−*μ*. Adding spin-orbit coupling *α*≠0 will generically lift these degeneracy lines and gap the spectrum, except if the degeneracy occurs at the four inversion-symmetric momenta [**k**_*ij*_=(1−*i*, 1−*j*)*π*/2 with *i*, *j*=±] where the spin-orbit coupling vanishes. The occurrence of these nodal points is a signature of transitions between phases characterized by different Chern numbers. The critical chemical potentials are therefore determined by equation (3) with **k**=**k**_*ij*_, which yields


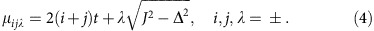


Around each of the nodal points in the three-dimensional **k**-*μ* parameter space, we can reduce the Hamiltonian to an effective two-band model and expand it to linear order in the deviations *δ***k** from **k**_*ij*_ and the deviations *δμ* from *μ*_*ijλ*_ yielding


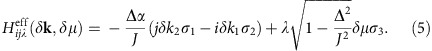


Here the Pauli matrices act on a subspace defined by the two bands that satisfy condition in equation (3). In the **k**-*μ* space, the Hamiltonian in equation (5) is a Weyl Hamiltonian that is characterized by a unit topological charge *i* × *j* × *λ*=±1. As the chemical potential ramps through a critical point at *μ*_*ijλ*_, the Chern number associated with the original Hamiltonian in equation (1) changes precisely by the value of the topological charge, or the total topological charges when multiple nodal points occur at the same *μ*. Therefore, by increasing *μ* and assuming 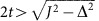, the system exhibits phases with the Chern numbers (see Supplementary Note 1)


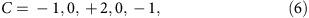


where we have neglected the trivial phases with *μ* falling outside the band width. Hence, with homogeneous magnetization, the superconductor may already exhibit Chern number equal to 2. In cases where magnetic impurities are spaced more sparsely, that is, if Λ* is a sublattice of Λ, even higher Chern numbers can be obtained.

**Figure 2:**
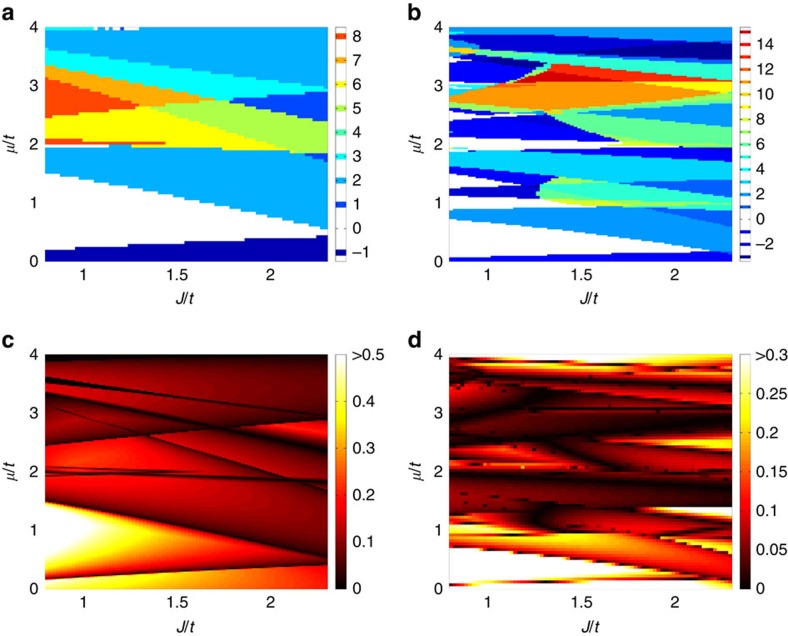
Phase diagrams in the dense impurity limit. (**a**,**b**) Chern numbers, and (**c**,**d**) ratios of the induced superconducting gap to the host superconducting gap, as functions of the chemical potential *μ* and the exchange field strength *J*, both in units of hopping strength *t*. (**a**) and (**c**), (**b**) and (**d**) correspond to the case of one magnetic adatom every 2 × 2, or 3 × 3 lattice sites, respectively. These phase diagrams are obtained by using the Hamiltonian in equation (1) with Δ=0.06*t* and *α*=0.1*t*; *μ* has been shifted such that *μ*=0 implies the chemical potential lying in the center between the two spin-split band bottoms.

We solved the Hamiltonian in equation (1) numerically for the case of one magnetic impurity every 2 × 2 and 3 × 3 plaquettes of the square lattice, and show the phase diagrams in Fig. 2. From the phase diagrams, we can read off three general features. First, at small chemical potentials around the band bottom, where the Fermi wavelengths are larger than or comparable to the lattice spacing of Λ*, the sequence of Chern numbers (−1, 0, +2) always occurs when *J*/*t* is not too large—this universal feature corresponds to the dense limit that we have discussed above; Second, at larger chemical potentials, more than two Fermi surfaces can exist in the reduced Brillouin zone defined by Λ* as the Fermi wavelengths are significantly smaller than the lattice spacing of Λ*, as a consequence higher Chern numbers can occur (for example, 8 in Fig. 2a and 15 in Fig. 2b) but the trade-off is an overall smaller induced gap; Third, the phases with different Chern numbers are generally separated by lines (in the 2D parameter space) defined by conditions similar to equation (4), and across each specific line the change of Chern numbers is a constant determined essentially the same way as in our preceding analysis.

### Dilute impurity limit

To better understand the dilute impurity limit, we complement our results on the lattice by a calculation in which we treat the underlying 2D superconductor in the continuum limit and consider sparsely distributed Shiba impurities that are arranged in a square lattice on top of it. This allows us to derive an effective two-band model for the hybridizing Shiba states. This effective Hamiltonian represents a chiral *p*-wave superconductor in the appropriate parameter regime.

The strategy of our derivation is inspired by the calculation for 1D Shiba chains of Pientka *et al*.^29^ (for details, see Methods and Supplementary Note 2). We start from a 4 × 4 Bogoliubov-de Gennes (BdG) Hamiltonian


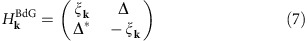


that acts on 4-spinor valued wave functions 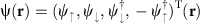, 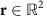, with *ξ*_**k**_=**k**^2^/(2*m*)−*μ*+*α*(*k*_1_*σ*_2_−*k*_2_*σ*_1_). In addition, the magnetic impurities are represented by the Hamiltonian


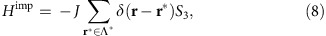


where *S*_3_=*σ*_3_⊗*τ*_0_, with *τ*_0_ the identity matrix acting on particle-hole space. If we restrict the wave-function Ψ(**r**) to the locations r*∈Λ* of the impurities, we obtain the self-consistency equation


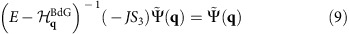


for the Fourier transforms 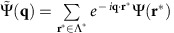, where the momentum **q**∈[0,2*π*)^2^ now belongs to the Λ* Brillouin zone and we have set the impurity spacing to unity.

We need two more steps to reduce equation (9) to an effective two-band model for the Shiba states, assuming they are deep in the superconducting gap and dilute compared with the Fermi wavelength. First, the left-hand side is expanded to linear order in the energy *E* to cast the equation in the form of the time-independent Schroedinger equation. Second, we project the effective Hamiltonian into the eigenstates of an isolated Shiba impurity on every site, given by 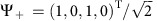 and 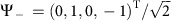 (ref. 29). We obtain the effective two-band Hamiltonian


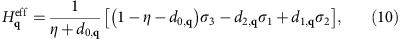


where


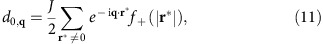



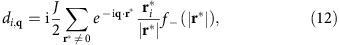


are defined in terms of the functions


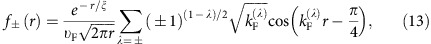


In the equation above, *ξ* is the coherence length of the 2D superconductor (without the magnetic impurities), 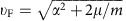 is its Fermi velocity, 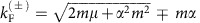 are the Fermi wave-vectors for its two spin-split bands, and 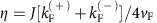 is a dimensionless parameter. In the deep Shiba limit in which the projection in the states *Ψ*_+_ and *Ψ*_−_ is justified, we have *η*∼1.

**Figure 3:**
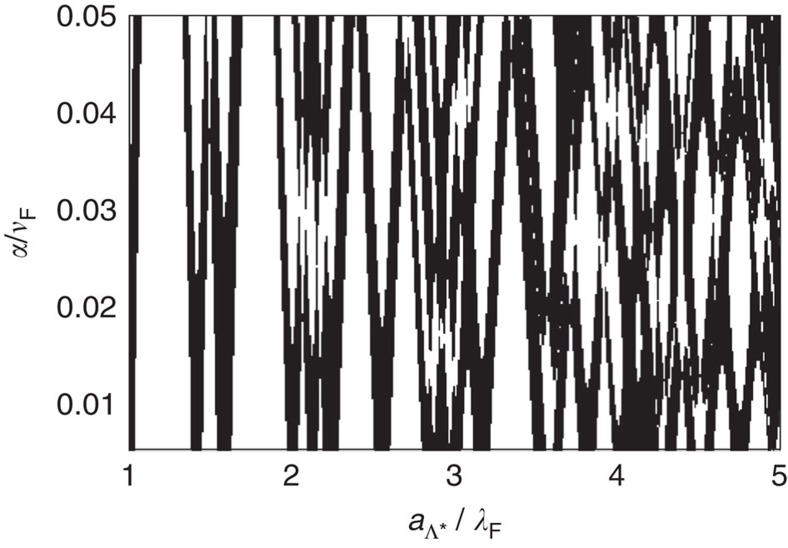
Phase diagram in the dilute impurity limit. Parity of the Chern number (white for even and black for odd) for the Bogoliubov-de Gennes band structure of an *s*-wave superconductor decorated with dilute Shiba impurities, following equation (14). Here the spin-orbit coupling strength *α* is scaled by the Fermi velocity *v*_F_, and the lattice spacing of the Shiba impurities *a*_Λ*_ is scaled by the Fermi wavelength *λ*_F_. Both *v*_F_ and *λ*_F_ are taken in the limit *α*→0 and we have assumed the superconducting coherence length *ξ*=30*λ*_F_.

The Hamiltonian in equation (10) represents the effective superconductor formed by the Shiba bound states within the gap of the underlying *s*-wave superconductor. Similar to the case of the effective two-band model in equation (5), this Hamiltonian can have nodal points in **k**-*μ*-space at which the Chern number changes. However, unlike in equation (5), the nodes can occur at any point in the Brillouin zone, making an analytic treatment intractable. In addition, the validity of equation (10) requires a self-consistency that permits the low-energy expansion of equation (9). Therefore, instead of computing the Chern numbers in an extended parameter space (for examples of Chern numbers along several linecuts of the parameter space, see Supplementary Fig. 1), we focus on information that can be obtained at special points of the Brillouin zone at infinitesimal energy. To that end, observe that the Hamiltonian in equation (10) has *C*_4_ rotational symmetry. Thus, any gap closing at points other than the *C*_4_-symmetric momenta **k**=(0, 0) and **k**=(*π*, *π*) changes the Chern number by an even integer due to the symmetry-imposed multiplicity of the nodal points. By expanding the Hamiltonian into Dirac form around the *C*_4_-symmetric momenta, we obtain the expression (see Supplementary Note 2)


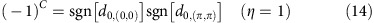


for the parity of the Chern number. The numerical evaluation of this equation is shown in Fig. 3 in the form of a phase diagram.

### Helical magnetic order

We have also performed calculations for magnetic orders other than simple ferromagnetism. In particular, the case where the magnetic configurations corresponds to 2D helices is related to previous studies on 1D helices^31^,^32^,^33^,^34^,^35^,^36^,^37^,^38^,^39^,^40^. We obtained criteria for such a system to be fully gapped by proximity effect, and found that the fully-gapped superconducting phases can be generically topologically nontrivial. The results and phase diagrams are presented in Supplementary Note 3 and Supplementary Figs 2–4.

### Material proposal

**Figure 4:**
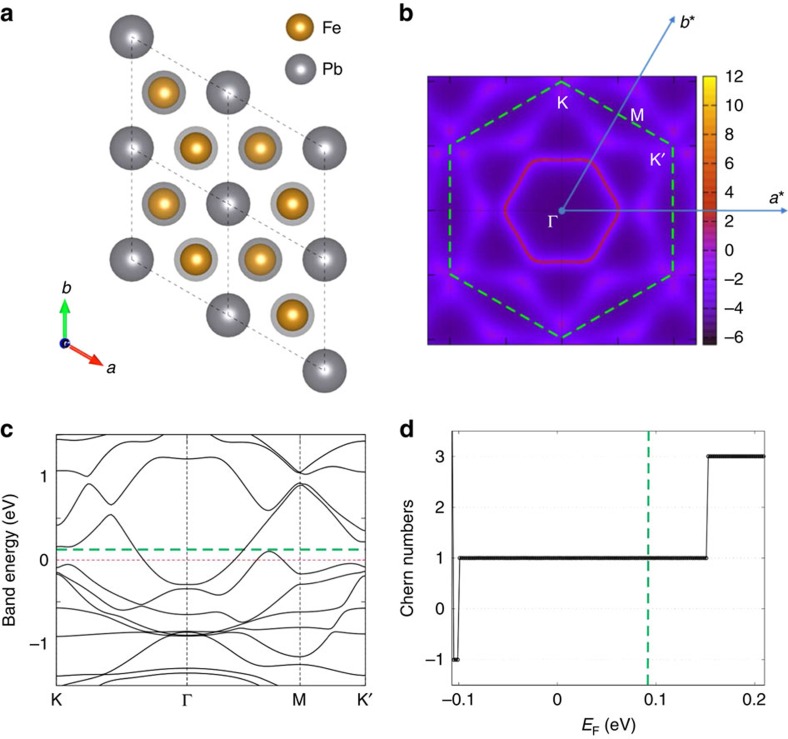
Fe adatoms on Pb (111) surface. Electronic structure obtained from density-functional-theory (DFT) calculations. (**a**) The atomic configuration: Fe adatoms form a buckled a honeycomb lattice on the Pb (111) surface. (**b**) Fermi surface of the Fe *d*-orbitals at energy *E*_F_=0.09 eV, where the Wigner–Seitz cell (dashed line) and the primitive vectors (arrowed lines) for the reciprocal lattice are indicated. A single Fermi surface is found around the Γ point in this case. (**c**) DFT band structure of the Fe *d*-orbitals with spin-orbit coupling. (**d**) Chern number of the Bogoliubov-de Gennes bands, as a function of Fermi energy, when *s*-wave superconductivity (Δ=0.01 eV in this calculation) is introduced in the system. The green dashed lines in both (**c**) and (**d**) mark the same energy corresponding to the Fermi surface given in (**b**).

We complement our simple model considerations with a specific material proposal to realize a superconductor with nonzero Chern numbers. For that, we consider transition metal atoms, in particular Fe, deposited on the (111) surface of Pb, a strong type-II superconductor. The same combination of materials, but a different surface of Pb, was used in the experimental realization for the 1D *p*-wave superconductor^25^. The Pb atoms on the (111) surface form a triangular lattice. Through *ab-initio* calculations that assume a ferromagnetic alignment of the Fe magnetic moments and include spin-orbit coupling, we compared the relaxation energies for various densities and arrangements of Fe adatoms on the Pb (111) surface, and found that a deposition with one Fe atom in each triangular plaquette is particularly favorable (see Supplementary Figs 5 and 6). In this case, the Fe atoms form a honeycomb lattice in which the atoms sit at different heights in each sublattice (see Fig. 4a). We further performed DFT calculations of the electronic structure. The resulting (not spin-degenerate) Fermi surface and band structure restricted to the Fe *d*-orbitals is shown in Fig. 4b,c. Critically, we find an odd number of Fermi surfaces (for examples of the Fermi surfaces at several Fermi energies, see Supplementary Fig. 7). The Chern number of the corresponding BdG Hamiltonian is indeed nonzero over a large range of the chemical potential (see Fig. 4d).

## Discussion

In conclusion, we have proposed a versatile platform for realizing chiral superconductors in 2D. We have obtained analytically the topological phase diagram (Chern number and gaps of the superconductor) of the dilute and dense limit, and numerically evaluated the phase diagram in the intermediate regime. We then showed through a more realistic *ab-initio* calculation that ferromagnetically ordered Fe atoms on the (111) surface of Pb in the dense limit could give rise to a chiral superconductor. To find flat islands of magnetic adatoms on the Pb substrate, however, is currently an experimental challenge because under standard growth conditions the magnetic adatoms tend to ball up instead of making flat islands on the (111) surface of Pb (see Supplementary Fig. 5).

The presence of a 2D chiral superconductor could be established experimentally by tunneling into the chiral Majorana modes, whose number is equal to the Chern number of the phase, and which would take place only on the edge of a 2D thin island of Fe on the surface of Pb. Such a technique has been used to image 1D topological edge states of bismuth bilayers in the absence of superconductivity^41^. Similar observations of quasiparticle edge modes inside the superconducting gap will be a strong signature of topological superconductivity proposed in this paper.

## Methods

### Effective Hamiltonian in the dilute impurity limit

We outline the general formalism by which an effective Hamiltonian for bound states of a Shiba lattice can be derived in the limit where the impurity spacing is large compared with the spacial extend of the bound states of an isolated impurity. We want to derive the low-energy effective theory for a Hamiltonian of the general form


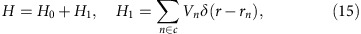


where *H*_0_ is the original superconducting BdG Hamiltonian which is gapped (≈Δ) around zero energy, and *H*_1_ is the Hamiltonian for a collection of delta-function impurities at positions *r*_*n*_, where *n* takes values in a set *c*, for example, a lattice. Here, *V*_*n*_ are matrices associated with the local degrees of freedom (such as spin and particle-hole) which can induce in-gap states. We implicitly keep *r* as a *d*-dimensional vector, so that the formalism is applicable for systems in any dimension *d* (the same applies to *r*_*n*_, *n*, *m*, *k*, *q*, *R* and *n*_*b*_ below).

We start with the Schrödinger equation for bound state wave functions Ψ


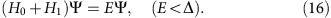


It follows that


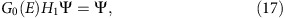


where *G*_0_(*E*)=(*E*−*H*_0_)^−1^ is the Green function.

Because *H*_1_ is composed of delta-functions for a small set *c*, *G*_0_*H*_1_ is nonzero only in the columns corresponding to *c*, thus equation 17 is equivalent to


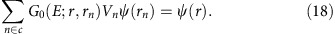


In the simplest case, if *c* contains only one single point, labelled by 0, then equation 18 implies


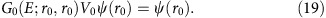


The energy of the excitations can be obtained by solving


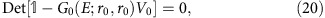



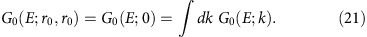


In more complicated cases, the following equation, again implied by equation 18, can serve as the starting point to extract an effective Hamiltonian


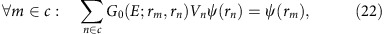



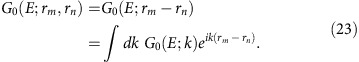


In addition, if ∀*n*: *V*_*n*_=*V*_0_ and *r*_*n*_=*nR* (*n*∈
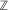
^*d*^ with *d* the dimension), equation (22) can be transformed to *k*-space:


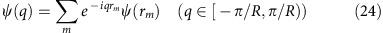



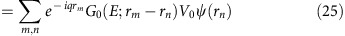



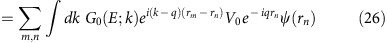



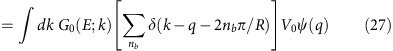



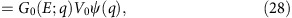


where


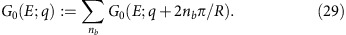


*n*_*b*_ can be interpreted as the band index when the *k*-space is folded into the Brillouin zone defined by [−*π*/*R*, *π*/*R*). The application of this formalism to the Hamiltonian in equation (7) is detailed in Supplementary Note 2 and results in the Hamiltonian in equation (10).

### First principle calculations

We performed electronic structure calculations within the DFT formalism as implemented in the Vienna *ab initio* simulation package^42^. We used the all-electron projector augmented wave^43^,^44^ basis sets with the generalized gradient approximation of Perdew, Burke and Ernzerhof^45^ to the exchange correlation potential. The Hamiltonian contains scalar relativistic corrections, and the spin-orbit coupling was taken into account by the second variation method^46^.

In this work, we chose the host superconductor to be Pb thin film with a (111) surface, and consider different transition metal adatoms. We started by finding the stable configurations of the adatoms on top of the Pb surface. To this end we have compared the relaxation energy (per atom) for an extensive collection of possible configurations, four of which are shown in Supplementary Fig. 5. Based on this energetic consideration and for simplicity, we adopted the configuration with two adatoms per Pb unit cell (highlighted in Supplementary Fig. 5) in our following simulations. When the transition metal element is chosen to be Fe, the details of the configuration are shown in Supplementary Fig. 6. In this configuration, two species of Fe atoms form a buckled honeycomb structure which gains bonding energy due to the short nearest-neighbor distance.

To simulate the composite system and consider the effect of Pb, we used six layers of Pb atoms as substrate in the relaxation calculations with roughly 15 Å vacuum space, taking into account spin-orbital coupling and the ferromagnetic alignment of the Fe moments. We performed DFT calculations with the abovementioned stable configuration. Based on the DFT calculations, we constructed the maximally localized Wannier functions^47^,^48^ for Fe, and obtained a tight-binding model with a band structure that agrees well with the DFT result. We then used the tight-binding model and added a small *s*-wave superconducting pairing term to it. We computed the Chern numbers of the thus-obtained Bogoliubov-deGennes Hamiltonian to be nonvanishing, as shown in Fig. 4. For completeness, we present a few more Fermi surfaces with different values of *E*_F_ in Supplementary Fig. 7 to complement Fig. 4.

## Additional information

**How to cite this article:** Li, J. *et al*. Two-dimensional chiral topological superconductivity in Shiba lattices. *Nat. Commun.* 7:12297 doi: 10.1038/ncomms12297 (2016).

## Supplementary Material

Supplementary Information Supplementary Figures 1-7, Supplementary Notes 1-3 and Supplementary References.

## References

[b1] Moore G. & Read N. Nonabelions in the fractional quantum hall effect. Nucl. Phys. B 360, 362–396 (1991).

[b2] Read N. & Green D. Paired states of fermions in two dimensions with breaking of parity and time-reversal symmetries and the fractional quantum Hall effect. Phys. Rev. B 61, 10267 (2000).

[b3] Ivanov D. A. Non-abelian statistics of half-quantum vortices in p-wave superconductors. Phys. Rev. Lett. 86, 268 (2001). 11177808 10.1103/PhysRevLett.86.268

[b4] Kitaev A. Y. Fault-tolerant quantum computation by anyons. Ann. Phys. 303, 2–30 (2003).

[b5] Nayak C., Simon S., Stern A., Freedman M. & Das Sarma S. Non-abelian anyons and topological quantum computation. Rev. Mod. Phys. 80, 1083–1159 (2008).

[b6] Tewari S., Das Sarma S., Nayak C., Zhang C. & Zoller P. Quantum computation using vortices and majorana zero modes of a *p*_*x*_+*ip*_*y*_ superfluid of fermionic cold atoms. Phys. Rev. Lett. 98, 010506 (2007). 17358465 10.1103/PhysRevLett.98.010506

[b7] Das Sarma S., Nayak C. & Tewari S. Proposal to stabilize and detect half-quantum vortices in strontium ruthenate thin films: non-abelian braiding statistics of vortices in a *p*_*x*_+*ip*_*y*_ superconductor. Phys. Rev. B 73, 220502 (2006).

[b8] Mackenzie A. P. & Maeno Y. The superconductivity of Sr_2_RuO_4_ and the physics of spin-triplet pairing. Rev. Mod. Phys. 75, 657–712 (2003).

[b9] Raghu S., Kapitulnik A. & Kivelson S. A. Hidden quasi-one-dimensional superconductivity in Sr_2_RuO_4_. Phys. Rev. Lett. 105, 136401 (2010). 21230791 10.1103/PhysRevLett.105.136401

[b10] Wang Q. H. . Theory of superconductivity in a three-orbital model of Sr_2_RuO_4_. EPL (Europhys. Lett.) 104, 17013 (2013).

[b11] Scaffidi T., Romers J. C. & Simon S. H. Pairing symmetry and dominant band in Sr_2_RuO_4_. Phys. Rev. B 89, 220510 (2014).

[b12] Sato M., Takahashi Y. & Fujimoto S. Non-abelian topological order in s-wave superfluids of ultracold fermionic atoms. Phys. Rev. Lett. 103, 020401 (2009). 19659186 10.1103/PhysRevLett.103.020401

[b13] Lee P. A. Proposal for Creating a Spin-polarized *p*_*x*_+*ip*_*y*_ State and Majorana Fermions. Preprint at http://arxiv.org/abs/0907.2681 (2009).

[b14] Sau J. D., Lutchyn R. M., Tewari S. & Das Sarma S. Generic new platform for topological quantum computation using semiconductor heterostructures. Phys. Rev. Lett. 104, 040502 (2010). 20366693 10.1103/PhysRevLett.104.040502

[b15] Alicea J. Majorana fermions in a tunable semiconductor device. Phys. Rev. B 81, 125318 (2010).

[b16] Kitaev A. Y. Unpaired Majorana fermions in quantum wires. Physics-Uspekhi 44, 131–136 (2001).

[b17] Lutchyn R. M., Sau J. D. & Das Sarma S. Majorana fermions and a topological phase transition in semiconductor-superconductor heterostructures. Phys. Rev. Lett. 105, 077001 (2010). 20868069 10.1103/PhysRevLett.105.077001

[b18] Oreg Y., Refael G. & von Oppen F. Helical liquids and majorana bound states in quantum wires. Phys. Rev. Lett. 105, 177002 (2010). 21231073 10.1103/PhysRevLett.105.177002

[b19] Potter A. C. & Lee P. A. Multichannel generalization of Kitaev's majorana end states and a practical route to realize them in thin films. Phys. Rev. Lett. 105, 227003 (2010). 21231416 10.1103/PhysRevLett.105.227003

[b20] Alicea J., Oreg Y., Refael G., von Oppen F. & Fisher M. P. A. Non-abelian statistics and topological quantum information processing in 1D wire networks. Nat. Phys. 7, 412–417 (2011).

[b21] Halperin B. I. . Adiabatic manipulations of Majorana fermions in a three-dimensional network of quantum wires. Phys. Rev. B 85, 144501 (2012).

[b22] Stanescu T. D. & Tewari S. Majorana fermions in semiconductor nanowires: fundamentals, modeling, and experiment. J. Phys.: Condens. Matter 25, 233201 (2013). 23665894 10.1088/0953-8984/25/23/233201

[b23] Mourik V. . Signatures of majorana fermions in hybrid superconductor-semiconductor nanowire devices. Science 336, 1003–1007 (2012). 22499805 10.1126/science.1222360

[b24] Li J. . Topological superconductivity induced by ferromagnetic metal chains. Phys. Rev. B 90, 235433 (2014).

[b25] Nadj-Perge S. . Observation of Majorana fermions in ferromagnetic atomic chains on a superconductor. Science 346, 602–607 (2014). 25278507 10.1126/science.1259327

[b26] Yu L. Bound state in superconductors with paramagnetic impurities. Acta Phys. Sin. 21, 75–91 (1965).

[b27] Shiba H. Classical spins in superconductors. Prog. Theor. Phys. 40, 435–451 (1968).

[b28] Rusinov A. I. On the theory of gapless superconductivity in alloys containing paramagnetic impurities. Soviet Phys. JETP 29, 1101–1106 (1969).

[b29] Pientka F., Glazman L. I. & von Oppen F. Topological superconducting phase in helical Shiba chains. Phys. Rev. B 88, 155420 (2013).

[b30] Heimes A., Mendler D. & Kotetes P. Interplay of topological phases in magnetic adatom-chains on top of a Rashba superconducting surface. New. J. Phys. 17, 023051 (2015).

[b31] Choy T.-P., Edge J. M., Akhmerov A. R. & Beenakker C. W. J. Majorana fermions emerging from magnetic nanoparticles on a superconductor without spin-orbit coupling. Phys. Rev. B 84, 195442 (2011).

[b32] Nadj-Perge S., Drozdov I. K., Bernevig B. A. & Yazdani A. Proposal for realizing Majorana fermions in chains of magnetic atoms on a superconductor. Phys. Rev. B 88, 020407 (2013).

[b33] Nakosai S., Tanaka Y. & Nagaosa N. Two-dimensional p-wave superconducting states with magnetic moments on a conventional s-wave superconductor. Phys. Rev. B 88, 180503 (2013).

[b34] Klinovaja J., Stano P., Yazdani A. & Loss D. Topological superconductivity and Majorana Fermions in RKKY Systems. Phys. Rev. Lett. 111, 186805 (2013). 24237550 10.1103/PhysRevLett.111.186805

[b35] Braunecker B. & Simon P. Interplay between classical magnetic moments and superconductivity in quantum one-dimensional conductors: toward a self-sustained topological Majorana phase. Phys. Rev. Lett. 111, 147202 (2013). 24138267 10.1103/PhysRevLett.111.147202

[b36] Vazifeh M. M. & Franz M. Self-organized topological state with Majorana fermions. Phys. Rev. Lett. 111, 206802 (2013). 24289700 10.1103/PhysRevLett.111.206802

[b37] Pöyhönen K., Westström A., Röntynen J. & Ojanen T. Majorana states in helical Shiba chains and ladders. Phys. Rev. B 89, 115109 (2014).

[b38] Pientka F., Glazman L. I. & von Oppen F. Unconventional topological phase transitions in helical Shiba chains. Phys. Rev. B 89, 180505 (2014).

[b39] Kim Y., Cheng M., Bauer B., Lutchyn R. M. & Das Sarma S. Helical order in one-dimensional magnetic atom chains and possible emergence of Majorana bound states. Phys. Rev. B 90, 060401 (2014).

[b40] Li J., Neupert T., Bernevig B. A. & Yazdani A. Manipulating Majorana zero modes on atomic rings with an external magnetic field. Nat. Commun. 7, 10395 (2016). 26791080 10.1038/ncomms10395 PMC4735899

[b41] Drozdov I. K. . One-dimensional topological edge states of bismuth bilayers. Nat. Phys. 10, 664–669 (2014).

[b42] Kresse G. & Hafner J. *Ab initio* molecular dynamics for liquid metals. Phys. Rev. B 47, 558–561 (1993). 10.1103/physrevb.47.558 10004490

[b43] Blöchl P. Projector augmented-wave method. Phys. Rev. B 50, 17953–17979 (1994). 10.1103/physrevb.50.17953 9976227

[b44] Kresse G. & Joubert D. From ultrasoft pseudopotentials to the projector augmented-wave method. Phys. Rev. B 59, 1758–1775 (1999).

[b45] Perdew J., Burke K. & Ernzerhof M. Generalized gradient approximation made simple. Phys. Rev. Lett. 77, 3865–3868 (1996). 10062328 10.1103/PhysRevLett.77.3865

[b46] Koelling D. D. & Harmon B. N. A technique for relativistic spin-polarised calculations. J. Phys. C 10, 3107–3114 (1977).

[b47] Marzari N. & Vanderbilt D. Maximally localized generalized Wannier functions for composite energy bands. Phys. Rev. B 56, 12847–12865 (1997).

[b48] Souza I., Marzari N. & Vanderbilt D. Maximally localized Wannier functions for entangled energy bands. Phys. Rev. B 65, 035109 (2001).

