# Supplementary material for: Two-dimensional chiral topological superconductivity in Shiba lattices
Source: Nat Commun. 2016 Jul 28;7:12297. doi: 10.1038/ncomms12297 (PMC4974475; doi:10.1038/ncomms12297)
Supplement: Supplementary Information — Supplementary Figures 1-7, Supplementary Notes 1-3 and Supplementary References. [file ncomms12297-s1.pdf]

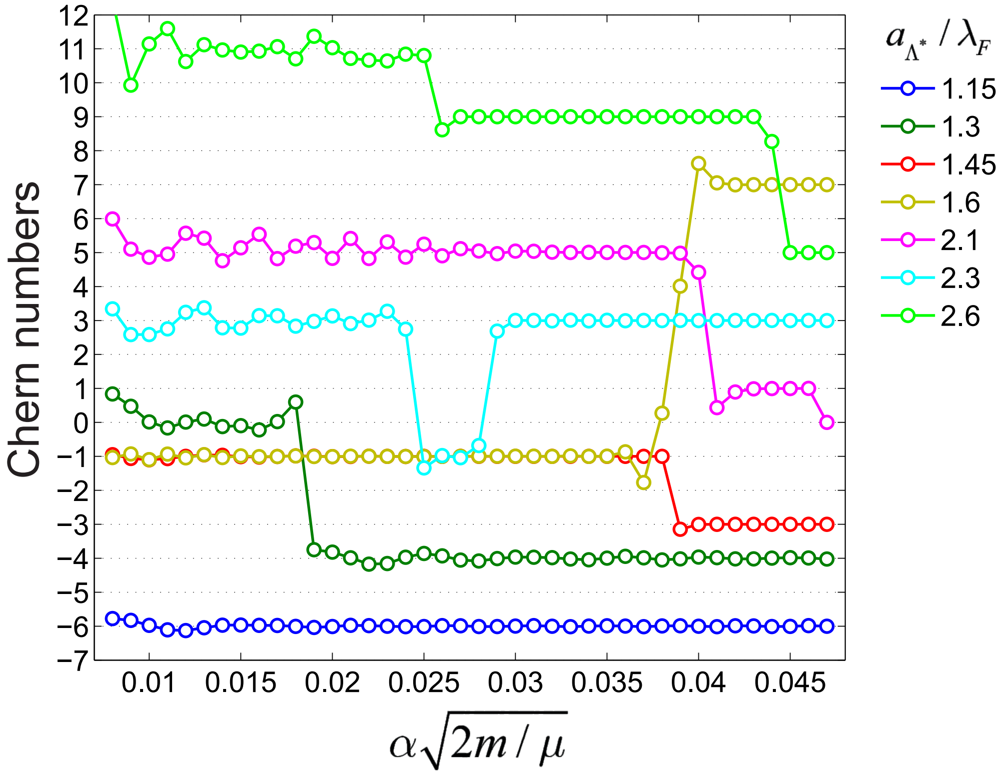

Supplementary Figure 1: Topology of Shiba bands of a ferromagnetic dilute impurity lattice. Chern numbers computed straightforwardly from Hamiltonian of Supplementary Equation (98) (with $\xi = 10\lambda_F$) as a function of spin-orbit coupling strength, for several values of lattice spacing (in units of Fermi wavelength λ_{F}) of Shiba impurities. The ill quantization of some of the Chern numbers is due to errors in numerical integrations. Note that the symbols here are dimensional to be consistent with the main text.

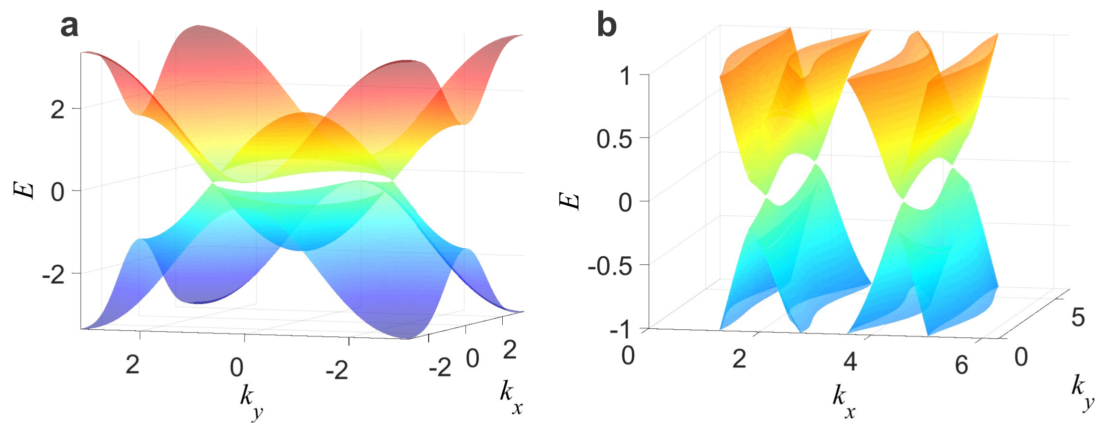

Supplementary Figure 2: Gapless low energy spectrum of helimagnetic impurity lattices. Examples of nodal superconductor band structures with (**a**) two Fermi points (see Supplementary Note 3 A), (**b**) four Fermi points (see Supplementary Note 3 B).

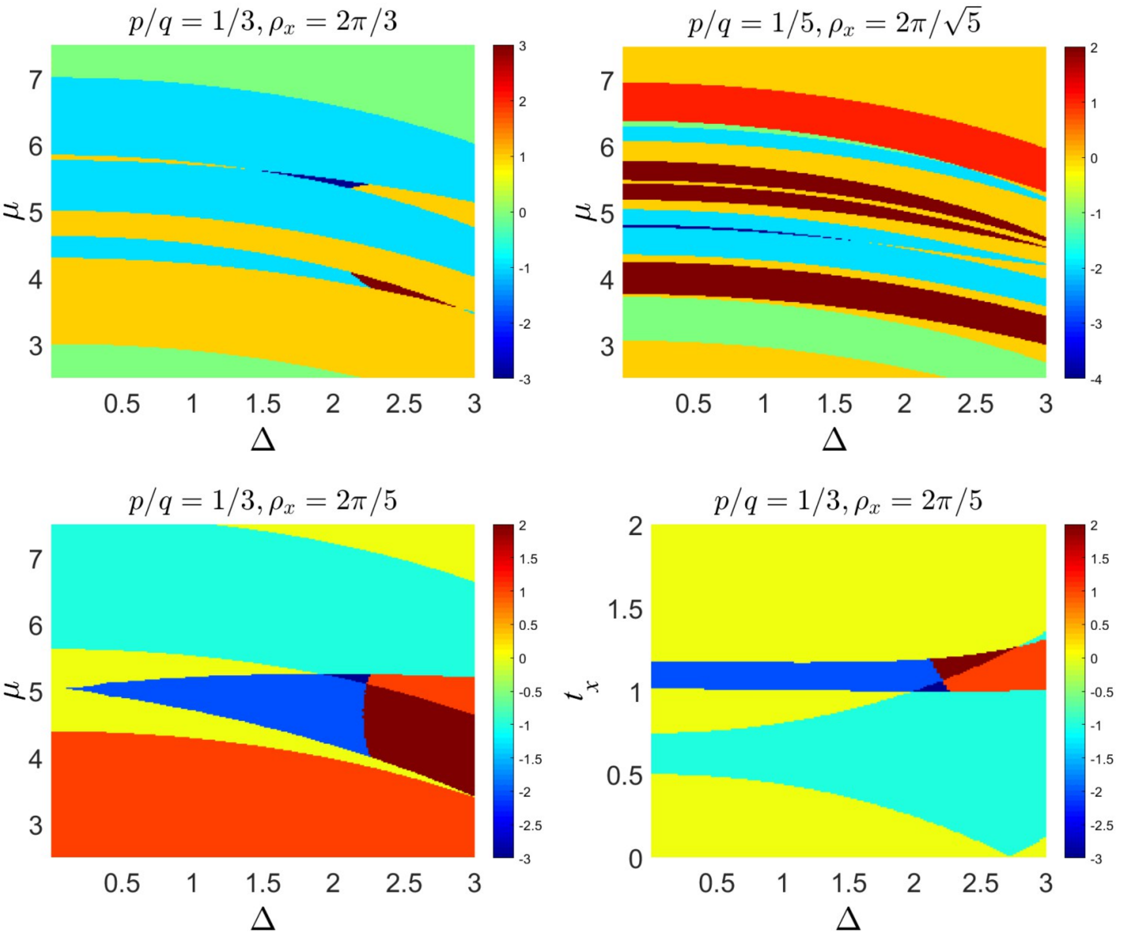

Supplementary Figure 3: Examples of the Chern number phase diagrams with different helical patterns. The color code corresponds to the Chern number C of the impurity bands.

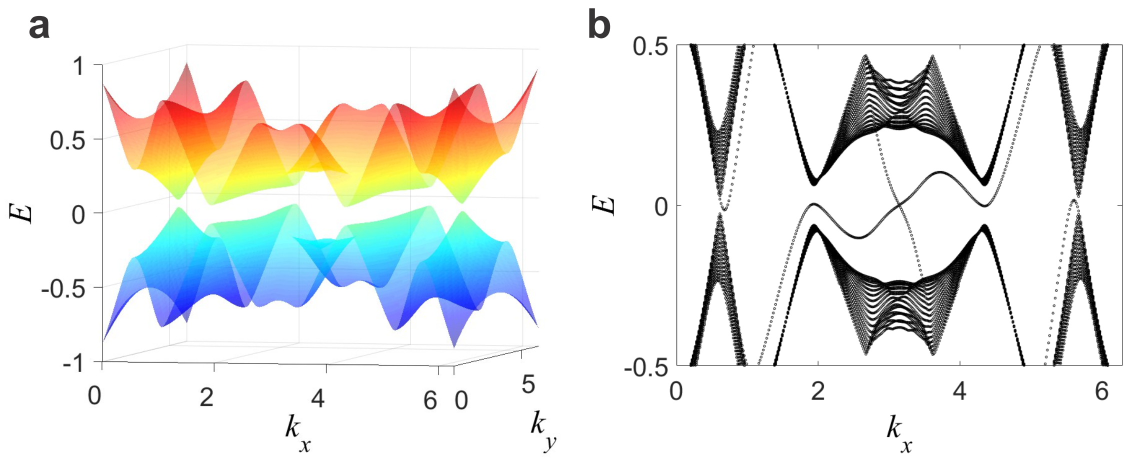

Supplementary Figure 4: Gapped low energy spectrum of helimagnetic impurity lattices: (**a**) low-energy bulk bands and (**b**) dispersion relations for an open-boundary strip. Here, $p/q = 1/3$, $\rho_x = 2\pi/3$, $\mu = 5.5$, $\Delta = 0.9$. The Chern number is -1 in this example.

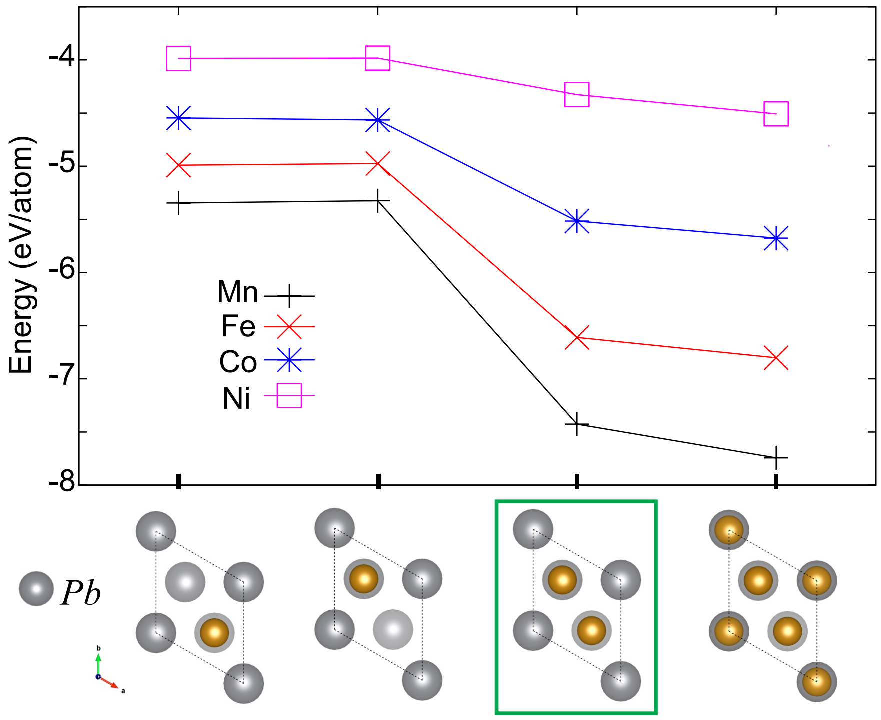

Supplementary Figure 5: Relaxation energy of different lattice configurations for different transition metal adatoms. The configuration adopted in our simulations is highlighted.

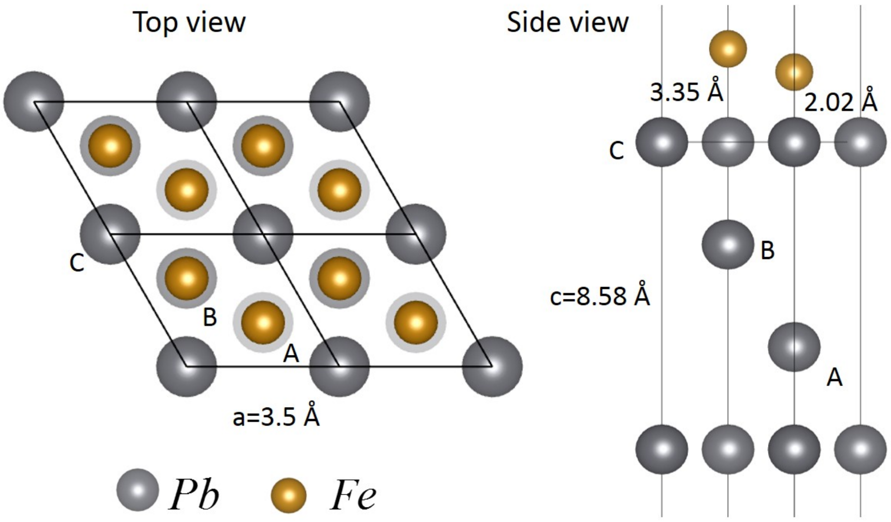

Supplementary Figure 6: Details of the atomic configuration adopted in our simulations. This configuration has a notable $C3$ symmetry.

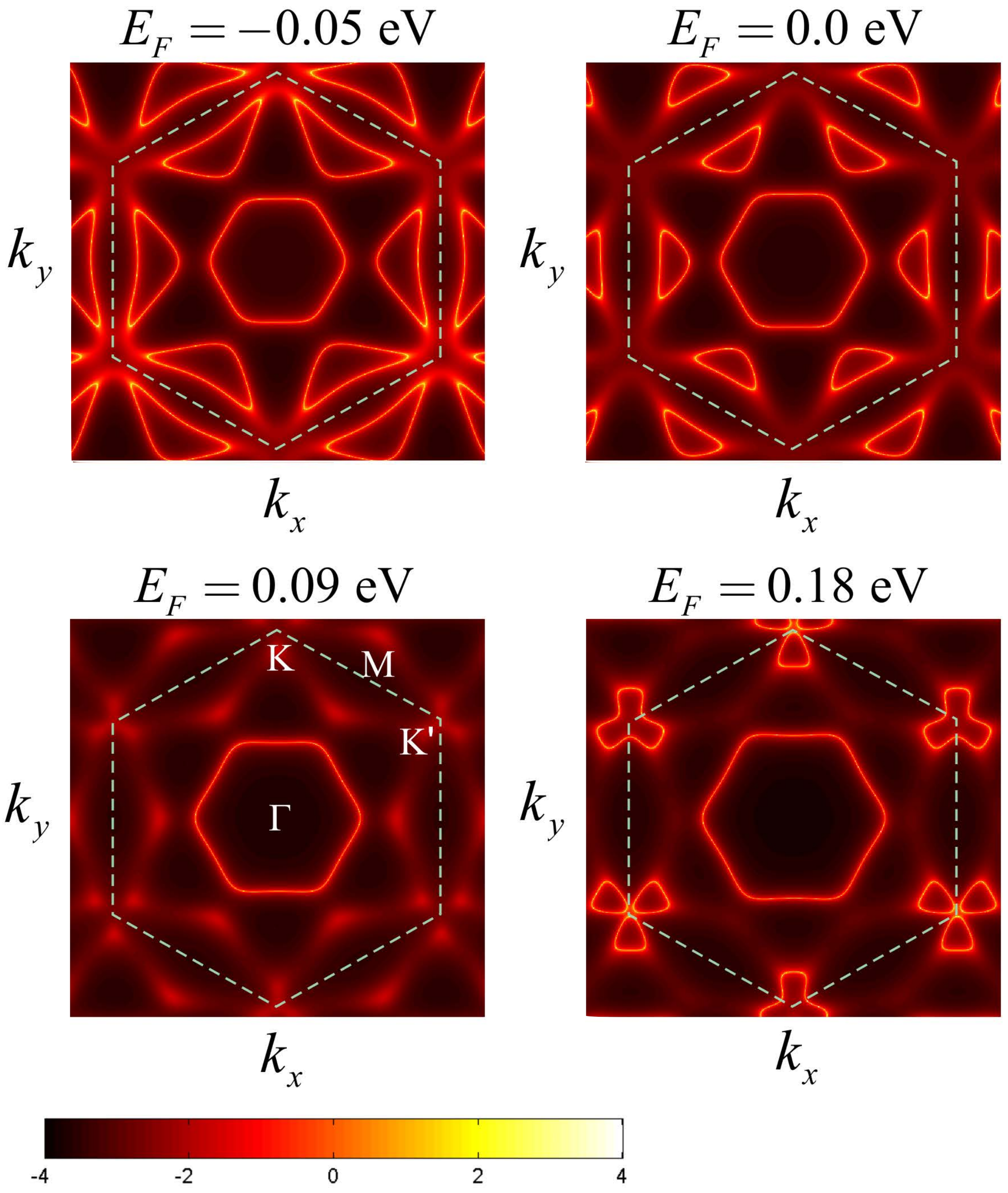

Supplementary Figure 7: Fermi surfaces with four values of E_{F} around 0. In the presence of a small (0.01eV) s-wave superconducting pairing potential, the Chern number corresponding to the superconducting phase with $E_{\mathrm{F}} = 0.18$ eV is 3, and the Chern numbers in the rest cases are all 1 (see main text Figure 4). The color scale represents $\log \rho(E_{\mathrm{F}}, k)$ in arbitrary units, where $\rho(E_{\mathrm{F}}, k)$ is the momentum-resolved density of states at the Fermi energy.

Supplementary Note 1. Analysis of topological phases in the dense impurity limit

In the dense impurity limit ($\Lambda = \Lambda^*$), Hamiltonian of Equation (1) in the main text has a simple form in momentum space

$$H = \sum_{\boldsymbol{k}} \boldsymbol{c}_{\boldsymbol{k}}^\dagger H_{\boldsymbol{k}} \boldsymbol{c}_{\boldsymbol{k}}, \qquad \boldsymbol{c}_{\boldsymbol{k}} = (c_{\boldsymbol{k},\uparrow}, c_{\boldsymbol{k},\downarrow}, c_{\boldsymbol{k},\downarrow}^\dagger, -c_{\boldsymbol{k},\uparrow}^\dagger)^T, \tag{1}$$

$$H_{\boldsymbol{k}} = \{[2t(\cos k_1 + \cos k_2) - \mu]\sigma_0 + 2\alpha(\sigma_1 \sin k_2 - \sigma_2 \sin k_1)\} \otimes \tau_3 + J\sigma_3 \otimes \tau_0 + \Delta\sigma_0 \otimes \tau_1, \tag{2}$$

where we have assumed the lattice constant $a = 1$, and the Pauli matrices σ_i and τ_i ($i = 0, 1, 2, 3$) correspond to spin and particle-hole degrees of freedom, respectively. The spectrum of $H_{\boldsymbol{k}}$ is given by

$$E(\boldsymbol{k}) = \pm\sqrt{J^2 + \Delta^2 + \varepsilon_{\boldsymbol{k}}^2 + \alpha_{\boldsymbol{k}}^2 \pm 2\sqrt{J^2(\Delta^2 + \varepsilon_{\boldsymbol{k}}^2) + \varepsilon_{\boldsymbol{k}}^2\alpha_{\boldsymbol{k}}^2}}, \tag{3}$$

where $\varepsilon_{\mathbf{k}} = 2t(\cos\, k_1 + \cos\, k_2) - \mu$ and $\alpha_{\boldsymbol{k}} = 2\alpha\sqrt{\sin^2 k_1 + \sin^2 k_2}$.

If $\alpha = 0$, degenerate zero eigenvalues of the above Hamiltonian occur where $\varepsilon_{\mathbf{k}} = \pm\sqrt{J^2 - \Delta^2}$, which generically appears as nodal lines in the momentum space (provided that solutions exist). After turning on $\alpha \neq 0$, the zero-energy solutions are in general removed except if they occur where $\sin^2 k_1 + \sin^2 k_2 = 0$, that is, at the four inversion-symmetric momenta [$\mathbf{k}_{ij} = (1 - i, 1 - j)\pi/2$ with $i, j = \pm 1$]. We will show next that the occurrences of these nodal points when the chemical potential μ is tuned with respect to the normal-state band structure signify transitions between topologically distinct superconducting phases.

First we notice that for a zero-energy nodal point to occur at $\mathbf{k}_{ij}$, the chemical potential must satisfy

$$\mu = \mu_{ij\lambda} := 2(i + j)t + \lambda\sqrt{J^2 - \Delta^2}, \qquad i, j, \lambda = \pm 1. \tag{4}$$

Around each of these points the original Hamiltonian of Supplementary Equation (1) can be expanded in the three-dimensional $\mathbf{k}$-μ space in the form of a (two-component) Weyl Hamiltonian. The topological difference between the two fully-gapped phases immediately above and below $\mu = \mu_{i,j,\lambda}$ is precisely given by the topological charge associated with the Weyl point, or, the sum of the topological charges in the presence of multiple Weyl points at the same μ. Explicitly, to linear order in the deviations ($\delta\mathbf{k}, \delta\mu$) from the nodal point ($\mathbf{k}_{ij}, \mu_{ij\lambda}$), the original Hamiltonian of

Supplementary Equation (1) becomes

$$H_{ij\lambda}^{\text{eff}}(\delta\mathbf{k},\delta\mu) = -\frac{\Delta\alpha}{J}\,(j\,\delta k_2\tilde{\sigma}_1 - i\,\delta k_1\tilde{\sigma}_2) + \lambda\sqrt{1-\frac{\Delta^2}{J^2}}\,\delta\mu\,\tilde{\sigma}_3, \tag{5}$$

where the Pauli matrices $\tilde{\sigma}_i$ act on a subspace defined by the two bands that are relevant at the specific nodal point. This effective Hamiltonian is obviously of a Weyl form and the associated topological charge (that is, the integrated Berry flux on a closed surface enclosing the Weyl point in the $\mathbf{k}$-μ space) is simply given by

$$n_{ij\lambda} = C_{ij\lambda}^{(+)} - C_{ij\lambda}^{(-)} = i \times j \times \lambda. \tag{6}$$

Here, we have used the fact that the difference of the Chern numbers ($C_{ij\lambda}^{(\pm)}$) corresponding to the fully-gapped 2D phases with μ immediately above ($+$) and below ($-$) $\mu_{ij\lambda}$, contributed by a single Weyl point at $(\mathbf{k}_{ij}, \mu_{ij\lambda})$, is equal to its topological charge

Supplementary Equation (6) allows us to infer the Chern numbers corresponding to all the topologically distinct phases when μ is tuned across the normal-state band width. To be specific, let us assume $2t > \sqrt{J^2-\Delta^2}$, so that the critical chemical potentials, defined in Supplementary Equation (4), are in the order $\mu_{---} < \mu_{--+} < (\mu_{-+-} = \mu_{+--}) < (\mu_{-++} = \mu_{+-+}) < \mu_{++-} < \mu_{+++}$. These six critical chemical potentials separate seven topologically distinct phases, with the first ($\mu < \mu_{---}$) and the last ($\mu > \mu_{+++}$) ones topologically trivial ($C = 0$ by definition). Thus, according to Supplementary Equation (6), the intermediate phases with increasing μ are characterized sequentially by Chern numbers

$$C = \ -1,\ 0,\ +2,\ 0,\ -1. \tag{7}$$

This sequence is confirmed by straightforward numerical calculations similar to what is shown in Figure 2 of the main text.

Supplementary Note 2. Derivation of the effective Hamiltonian in the dilute impurity limit

We apply the strategy outlined in the Methods section to the Hamiltonian in equation (7) of the main text in order to derive the effective theory in equation (10) of the main text. We start with the

BdG Hamiltonian

$$H_{BdG}(k) = \begin{pmatrix} \xi_k & \Delta_k \\ \Delta_k^\dagger & -\xi_k \end{pmatrix}, \tag{8}$$

$$\xi_k = \frac{\hbar^2}{2m^*}k^2 - \mu + \alpha(k_x\sigma_y - k_y\sigma_x)\,, \tag{9}$$

$$\Delta_k = \Delta_s\sigma_0 + \delta_p(k_x\sigma_y - k_y\sigma_x). \tag{10}$$

First we make the Hamiltonian dimensionless by defining

$$k_\mu = \sqrt{2m^*\mu/\hbar^2}, \quad \tilde{\boldsymbol{k}} = \boldsymbol{k}/k_\mu, \tag{11}$$

$$l_\alpha = \hbar^2/(2m^*\alpha), \quad \tilde{\alpha} = 1/(l_\alpha k_\mu), \tag{12}$$

$$l_p = \hbar^2/(2m^*\delta_p), \quad \Delta_p = 1/(l_p k_\mu), \tag{13}$$

$$\tilde{H} = H/\mu, \quad \tilde{\Delta}_s = \Delta_s/\mu, \tag{14}$$

and then omit the tildes, the bulk Hamiltonian becomes

$$\begin{aligned} H_{BdG}(\boldsymbol{k}) = \tau_z \otimes [(k^2-1)\sigma_0 + \alpha(k_x\sigma_y - k_y\sigma_x)] \\ +\tau_x \otimes [\Delta_s\sigma_0 + \Delta_p(k_x\sigma_y - k_y\sigma_x)]. \end{aligned} \tag{15}$$

In this dimensionless Hamiltonian, energies are measured in units of μ (assumed to be large compared with other energy scales), lengths (wave vectors) are measured in units of the Fermi wavelength (wave vector).

Next we go to the basis where the normal state Hamiltonian is diagonalized

$$\tilde{H}_{BdG}(k) = U(\varphi_k)^\dagger H_{BdG}(\boldsymbol{k})U(\varphi_k) \tag{16}$$

$$= \tau_z \otimes [(k^2-1)\sigma_0 + \alpha k\sigma_z] + \tau_x \otimes [\Delta_s\sigma_0 + \Delta_p k\sigma_z] \tag{17}$$

$$= [E_+(k)\tau_z + \Delta_+(k)\tau_x] \oplus [E_-(k)\tau_z + \Delta_-(k)\tau_x], \tag{18}$$

where

$$U(\varphi_k) = \tau_0 \otimes \frac{1}{\sqrt{2}} \begin{pmatrix} 1 & -1 \\ ie^{i\varphi_k} & ie^{i\varphi_k} \end{pmatrix}, \tag{19}$$

$$E_\pm(k) = k^2 - 1 \pm \alpha k, \tag{20}$$

$$\Delta_\pm(k) = \Delta_s \pm \Delta_p k. \tag{21}$$

According to the general formalism (see Methods section of the main text), we need to evaluate

$$G_0(E,\boldsymbol{r}) = \int d\boldsymbol{k}\; G_0(E,\boldsymbol{k}) e^{i\boldsymbol{k}\cdot\boldsymbol{r}} \tag{22}$$

$$= \int_0^\infty \frac{k\,dk}{2\pi} \int_0^{2\pi} \frac{d\varphi_k}{2\pi}\; U(\varphi_k)[E - \tilde{H}_{BdG}(k)]^{-1} U(\varphi_k)^\dagger e^{ikr\cos(\varphi_k-\varphi_r)} \tag{23}$$

$$= R(\varphi_r) \left[\int_0^{2\pi} \frac{d\varphi_k'}{2\pi}\; U(\varphi_k')\Gamma(E, r\cos\varphi_k') U(\varphi_k')^\dagger \right] R(\varphi_r)^\dagger, \tag{24}$$

where

$$\Gamma(E,x) = \int_0^\infty \frac{k\,dk}{2\pi} e^{ikx} [E - \tilde{H}_{BdG}(k)]^{-1}, \tag{25}$$

$$R(\varphi_r) = \tau_0 \otimes \begin{pmatrix} 1 & 0 \\ 0 & e^{i\varphi_r} \end{pmatrix}. \tag{26}$$

To evaluate $\Gamma(E,x)$, we first perform the integral

$$\Gamma_{b=\pm}(E,x) = \int_0^\infty \frac{k\,dk}{2\pi} e^{ikx} [E - E_b(k)\tau_z - \Delta_b(k)\tau_x]^{-1} \tag{27}$$

$$\simeq \rho_b \int dE_b\, e^{i[k_F^{(b)} + E_b/v_F]x}\, \frac{E + E_b\tau_z + \Delta_b\tau_x}{E^2 - E_b^2 - \Delta_b^2}, \tag{28}$$

where $v_F = 2k_0$ ($k_0 \equiv \sqrt{1+\alpha^2/4}$) is the Fermi velocity (the same for $\pm$ bands), $k_F^{(\pm)} = k_0 \mp \alpha/2$ is the Fermi wave vector for $+/-$-band, $\rho_b = k_F^{(b)}/2\pi v_F$ is the density of states for b-band at the Fermi energy, and $\Delta_b = \Delta_b(k_F^{(b)})$ is the pairing gap at the Fermi energy. Following Pientka et al. [1], we have

$$\int dE_b\, e^{iE_b x/v_F}\, \frac{1}{E_b^2 + a^2} = \frac{\pi}{a} e^{-a|x|/v_F}, \tag{29}$$

$$\int dE_b\, e^{iE_b x/v_F}\, \frac{E_b}{E_b^2 + a^2} \frac{\omega_D^2}{E_b^2 + \omega_D^2}$$

$$= \frac{i\pi\omega_D^2}{\omega_D^2 - a^2}\, \mathrm{sgn}(x) (e^{-a|x|/v_F} - e^{-\omega_D|x|/v_F}), \tag{30}$$

where $a = \sqrt{\Delta_b^2 - E^2}$ is a short-hand notation, and ω_D is the Debye frequency which will be sent to $+\infty$ in the end. Then

$$\Gamma_b = \gamma_{0b} + \gamma_{1b}\tau_x + \gamma_{3b}\tau_z, \tag{31}$$

$$\Gamma = \Gamma_+ \oplus \Gamma_- = \begin{pmatrix} \gamma_{0+} + \gamma_{3+} & 0 & \gamma_{1+} & 0 \\ 0 & \gamma_{0-} + \gamma_{3-} & 0 & \gamma_{1-} \\ \gamma_{1+} & 0 & \gamma_{0+} - \gamma_{3+} & 0 \\ 0 & \gamma_{1-} & 0 & \gamma_{0-} - \gamma_{3-} \end{pmatrix}, \tag{32}$$

where

$$\gamma_{0b} = -\pi\rho_b \frac{E}{\sqrt{\Delta_b^2 - E^2}} e^{ik_F^{(b)}x - \sqrt{\Delta_b^2 - E^2}|x|/v_F}, \tag{33}$$

$$\gamma_{1b} = -\pi\rho_b \frac{\Delta_b}{\sqrt{\Delta_b^2 - E^2}} e^{ik_F^{(b)}x - \sqrt{\Delta_b^2 - E^2}|x|/v_F}, \tag{34}$$

$$\gamma_{3b} = -\pi\rho_b \frac{i\omega_D^2}{\omega_D^2 + E^2 - \Delta_b^2} \operatorname{sgn}(x) e^{ik_F^{(b)}x}(e^{-\sqrt{\Delta_b^2 - E^2}|x|/v_F} - e^{-\omega_D|x|/v_F}). \tag{35}$$

Next we find

$$U(\varphi_k)\,\Gamma\,U(\varphi_k)^\dagger = \frac{1}{2}\begin{pmatrix} A_+ & -ie^{-i\varphi_k}A_- & C_+ & -ie^{-i\varphi_k}C_- \\ ie^{i\varphi_k}A_- & A_+ & ie^{i\varphi_k}C_- & C_+ \\ C_+ & -ie^{-i\varphi_k}C_- & B_+ & -ie^{-i\varphi_k}B_- \\ ie^{i\varphi_k}C_- & C_+ & ie^{i\varphi_k}B_- & B_+ \end{pmatrix}, \tag{36}$$

where

$$A_\pm = (\gamma_{0+} + \gamma_{3+}) \pm (\gamma_{0-} + \gamma_{3-}), \tag{37}$$

$$B_\pm = (\gamma_{0+} - \gamma_{3+}) \pm (\gamma_{0-} - \gamma_{3-}), \tag{38}$$

$$C_\pm = \gamma_{1+} \pm \gamma_{1-}. \tag{39}$$

The integral Supplementary Equation (24) breaks down to the following basic one

$$I_l(z) = i^l \int_{-\pi/2}^{\pi/2} d\varphi\; e^{i(l\varphi + z\cos\varphi)} \quad (l = 0, \pm 1) \tag{40}$$

$$= i^l \int_{-\pi/2}^{\pi/2} d\varphi\; e^{il\varphi} \left[\sum_{n=-\infty}^{+\infty} i^n J_n(z) e^{in\varphi}\right] \quad \text{(Jacobi-Anger expansion)} \tag{41}$$

$$= \sum_{n=-\infty}^{+\infty} i^{l+n} J_n(z) \int_{-\pi/2}^{\pi/2} d\varphi\; e^{i(l+n)\varphi} \tag{42}$$

$$= \pi J_{-l}(z) + 2i \sum_{m=-\infty}^{+\infty} \frac{J_{2m+1-l}(z)}{2m+1}, \tag{43}$$

where $J_n(z)$ is the the n-th order Bessel function of the first kind. Expressed in terms of $I_l(z)$, we

have

$$\int_0^{2\pi} \frac{d\varphi_k}{2\pi}\, i^l e^{il\varphi_k} \gamma_{0b}(\cos\varphi_k) = -\frac{\rho_b}{2}\, \frac{E}{\sqrt{\Delta_b^2 - E^2}} \left[I_l(z_b) + (-1)^l I_l(-z_b^*) \right], \tag{44}$$

$$\int_0^{2\pi} \frac{d\varphi_k}{2\pi}\, i^l e^{il\varphi_k} \gamma_{1b}(\cos\varphi_k) = -\frac{\rho_b}{2}\, \frac{\Delta_b}{\sqrt{\Delta_b^2 - E^2}} \left[I_l(z_b) + (-1)^l I_l(-z_b^*) \right], \tag{45}$$

$$\int_0^{2\pi} \frac{d\varphi_k}{2\pi}\, i^l e^{il\varphi_k} \gamma_{3b}(\cos\varphi_k) \tag{46}$$

$$= -\frac{\rho_b}{2}\, \frac{i\omega_D^2}{\omega_D^2 + E^2 - \Delta_b^2} \left[I_l(z_b) - (-1)^l I_l(-z_b^*) - I_l(z_b') + (-1)^l I_l(-z_b'^*) \right], \tag{47}$$

$$z_b \equiv k_F^{(b)} r + i\sqrt{\Delta_b^2 - E^2} r/v_F, \quad z_b' \equiv k_F^{(b)} r + i\omega_D r/v_F. \tag{48}$$

First we look at the limit $r \to 0$, such that $z_b, z_b' \to 0$. In this case

$$I_0(z=0) = \pi, \quad I_{\pm 1}(z=0) = \pm 2i. \tag{49}$$

We find

$$G_0(E, \boldsymbol{r} \to 0) = R(\varphi_r)(\tilde{A}\, \tau_0 \otimes \sigma_0 + \tilde{C}\, \tau_x \otimes \sigma_0) R(\varphi_r)^\dagger \tag{50}$$

$$= \tilde{A}\, \tau_0 \otimes \sigma_0 + \tilde{C}\, \tau_x \otimes \sigma_0, \tag{51}$$

$$\tilde{A} \equiv \frac{1}{2} \int_0^{2\pi} \frac{d\varphi_k}{2\pi} (\gamma_{0+} + \gamma_{0-}) \Big|_{r\to 0} = (-\frac{\pi}{2}) \sum_b \frac{\rho_b E}{\sqrt{\Delta_b^2 - E^2}}, \tag{52}$$

$$\tilde{C} \equiv \frac{1}{2} \int_0^{2\pi} \frac{d\varphi_k}{2\pi} (\gamma_{1+} + \gamma_{1-}) \Big|_{r\to 0} = (-\frac{\pi}{2}) \sum_b \frac{\rho_b \Delta_b}{\sqrt{\Delta_b^2 - E^2}}. \tag{53}$$

For $r > 0$, we assume Re$(z) \gg |$Im$(z)|$ and Re$(z) \gg |n^2 - 1/4|$ (note that Re$(z) = \pm k_F r$, and Im$(z) = \sqrt{\Delta_b^2 - E^2} r/v_F$ or $\omega_D r/v_F$ in our integrals), by using the asymptotic form

$$J_n(z) \approx \sqrt{\frac{2}{\pi z}} \cos(z - n\pi/2 - \pi/4), \tag{54}$$

we obtain

$$I_l(z|\text{Re}(z) > 0) \approx \pi J_{-l}(z) + 2i\sqrt{\frac{2}{\pi z}} \sum_{m=-\infty}^{+\infty} \frac{\cos[z - (2m+1-l)\pi/2 - \pi/4]}{2m+1} \tag{55}$$

$$= \pi J_{-l}(z) + 2i\sqrt{\frac{2}{\pi z}} \sin(z + l\pi/2 - \pi/4) \sum_{m=-\infty}^{+\infty} \frac{(-1)^m}{2m+1} \tag{56}$$

$$= \sqrt{\frac{2\pi}{z}} e^{i(z - \pi/4 + l\pi/2)} = i^l \sqrt{\frac{2\pi}{z}} e^{i(z-\pi/4)}, \tag{57}$$

$$I_l(z|\text{Re}(z) < 0) = [I_{-l}(-z^*)]^* \approx i^l \left[\sqrt{\frac{2\pi}{-z^*}} e^{i(-z^* - \pi/4)} \right]^*. \tag{58}$$

Therefore (assuming $\omega_D \to +\infty$ after the integral)

$$G_0(E, \boldsymbol{r}) = \frac{1}{2} R(\varphi_r) \begin{pmatrix} \tilde{A}_+ - i\tilde{A}_-\sigma_y & \tilde{C}_+ - i\tilde{C}_-\sigma_y \\ \tilde{C}_+ - i\tilde{C}_-\sigma_y & \tilde{B}_+ - i\tilde{B}_-\sigma_y \end{pmatrix} R(\varphi_r)^\dagger, \tag{59}$$

where

$$\tilde{A}_+ \equiv \int_0^{2\pi} \frac{d\varphi_k}{2\pi} A_+ \tag{60}$$

$$\approx -(\frac{E}{\sqrt{\Delta_+^2 - E^2}} c_+ F_+ + \frac{E}{\sqrt{\Delta_-^2 - E^2}} c_- F_-) + (s_+ F_+ + s_- F_-), \tag{61}$$

$$\tilde{A}_- \equiv \int_0^{2\pi} \frac{d\varphi_k}{2\pi} i e^{i\varphi_k} A_- \tag{62}$$

$$\approx (\frac{E}{\sqrt{\Delta_+^2 - E^2}} s_+ F_+ - \frac{E}{\sqrt{\Delta_-^2 - E^2}} s_- F_-) + (c_+ F_+ - c_- F_-), \tag{63}$$

$$\tilde{B}_+ \equiv \int_0^{2\pi} \frac{d\varphi_k}{2\pi} B_+ \tag{64}$$

$$\approx -(\frac{E}{\sqrt{\Delta_+^2 - E^2}} c_+ F_+ + \frac{E}{\sqrt{\Delta_-^2 - E^2}} c_- F_-) - (s_+ F_+ + s_- F_-), \tag{65}$$

$$\tilde{B}_- \equiv \int_0^{2\pi} \frac{d\varphi_k}{2\pi} i e^{i\varphi_k} B_- \tag{66}$$

$$\approx (\frac{E}{\sqrt{\Delta_+^2 - E^2}} s_+ F_+ - \frac{E}{\sqrt{\Delta_-^2 - E^2}} s_- F_-) - (c_+ F_+ - c_- F_-), \tag{67}$$

$$\tilde{C}_+ \equiv \int_0^{2\pi} \frac{d\varphi_k}{2\pi} C_+ \tag{68}$$

$$\approx -\frac{\Delta_+}{\sqrt{\Delta_+^2 - E^2}} c_+ F_+ + \frac{\Delta_-}{\sqrt{\Delta_-^2 - E^2}} c_- F_-, \tag{69}$$

$$\tilde{C}_- \equiv \int_0^{2\pi} \frac{d\varphi_k}{2\pi} i e^{i\varphi_k} C_- \tag{70}$$

$$\approx \frac{\Delta_+}{\sqrt{\Delta_+^2 - E^2}} s_+ F_+ - \frac{\Delta_-}{\sqrt{\Delta_-^2 - E^2}} s_- F_-, \tag{71}$$

with

$$c_b \equiv \cos[k_F^{(b)} r - \pi/4], \quad s_b \equiv \sin[k_F^{(b)} r - \pi/4], \tag{72}$$

$$F_b \equiv \frac{\sqrt{2\pi}\rho_b \, e^{-\sqrt{\Delta_b^2 - E^2} r / v_F}}{\sqrt{k_F^{(b)} r}} = \sqrt{k_F^{(b)}} \, \frac{e^{-\sqrt{\Delta_b^2 - E^2} r / v_F}}{\sqrt{2\pi r} v_F}. \tag{73}$$

Now we assume $\Delta_+ = \Delta_- = \Delta$, focusing on pure singlet superconductivity, and ignore

$O(E^2/\Delta^2)$ terms (the deep-dilute-Shiba-state approximation). Then

$$G_0(E,\boldsymbol{r}) \approx \frac{E}{\Delta}\tau_0 \otimes \tilde{\alpha}(\boldsymbol{r}) + \tau_x \otimes \tilde{\alpha}(\boldsymbol{r}) + \tau_z \otimes \tilde{\beta}(\boldsymbol{r}), \tag{74}$$

$$\tilde{\alpha}(\boldsymbol{r}) = -\frac{1}{2}(c_+F_+ + c_-F_-) - \frac{i}{2}(s_+F_+ - s_-F_-)(\sigma_y \cos\varphi_r - \sigma_x \sin\varphi_r), \tag{75}$$

$$\tilde{\beta}(\boldsymbol{r}) = \frac{1}{2}(s_+F_+ + s_-F_-) - \frac{i}{2}(c_+F_+ - c_-F_-)(\sigma_y \cos\varphi_r - \sigma_x \sin\varphi_r), \tag{76}$$

$$F_b \approx \sqrt{k_F^{(b)}}\,\frac{e^{-r/\xi}}{\sqrt{2\pi r}v_F} \qquad (\xi \equiv v_F/\Delta). \tag{77}$$

For an FM Shiba lattice, assume the magnetization is along $\hat{z}$, we have [cf. equation (18) from the main text],

$$\sum_{\boldsymbol{r}'} G_0(E,\boldsymbol{r}-\boldsymbol{r}')(-J\sigma_z \otimes \tau_0)\psi(\boldsymbol{r}') = \psi(\boldsymbol{r}), \tag{78}$$

or,

$$\frac{E}{\Delta}\sum_{\boldsymbol{r}'} M_1(\boldsymbol{r}-\boldsymbol{r}')\psi(\boldsymbol{r}') = \sum_{\boldsymbol{r}'} M_0(\boldsymbol{r}-\boldsymbol{r}')\psi(\boldsymbol{r}'), \tag{79}$$

$$M_1(\delta\boldsymbol{r}=0) = \left(\frac{1}{2}\pi J \sum_b \rho_b\right)\tau_0 \otimes \sigma_z, \tag{80}$$

$$M_1(\delta\boldsymbol{r}\neq 0) = \left(\frac{1}{2}J\right)\tau_0 \otimes [\tilde{c}_+\sigma_z - \tilde{s}_-(\sigma_x\cos\varphi_r + \sigma_y\sin\varphi_r)], \tag{81}$$

$$M_0(\delta\boldsymbol{r}=0) = \mathbb{1} - \left(\frac{1}{2}\pi J \sum_b \rho_b\right)\tau_x \otimes \sigma_z, \tag{82}$$

$$\begin{aligned} M_0(\delta\boldsymbol{r}\neq 0) = \left(\frac{1}{2}J\right)\big\{&-\tau_x \otimes [\tilde{c}_+\sigma_z - \tilde{s}_-(\sigma_x\cos\varphi_r + \sigma_y\sin\varphi_r)] \\ &+ \tau_z \otimes [\tilde{s}_+\sigma_z + \tilde{c}_-(\sigma_x\cos\varphi_r + \sigma_y\sin\varphi_r)]\big\}, \end{aligned} \tag{83}$$

where

$$\rho_\pm \equiv k_F^{(\pm)}/2\pi v_F, \tag{84}$$

$$\tilde{c}_\pm \equiv c_+F_+ \pm c_-F_- = \frac{e^{-r/\xi}}{\sqrt{2\pi r}v_F}\left\{\sqrt{k_F^{(+)}}\cos[k_F^{(+)}r - \pi/4] \pm \sqrt{k_F^{(-)}}\cos[k_F^{(-)}r - \pi/4]\right\}, \tag{85}$$

$$\tilde{s}_\pm \equiv s_+F_+ \pm s_-F_- = \frac{e^{-r/\xi}}{\sqrt{2\pi r}v_F}\left\{\sqrt{k_F^{(+)}}\sin[k_F^{(+)}r - \pi/4] \pm \sqrt{k_F^{(-)}}\sin[k_F^{(-)}r - \pi/4]\right\}. \tag{86}$$

In the limit where the Shiba impurities are ultimately dilute, Supplementary Equation (79)

becomes

$$\frac{E}{\Delta}\psi(\boldsymbol{r}) = \left(\frac{1}{\eta}\tau_0 \otimes \sigma_z - \tau_x \otimes \sigma_0\right)\psi(\boldsymbol{r}), \tag{87}$$

$$\eta \equiv \frac{1}{2}\pi J \sum_b \rho_b = \frac{J}{2v_F}\sqrt{1+\frac{\alpha^2}{4}}. \tag{88}$$

Since we have already assumed the deep Shiba state limit, where $\eta \sim 1$, we find the two low energy states

$$E_\pm = \pm\Delta\left(\frac{1}{\eta} - 1\right), \quad \psi_+ = \frac{1}{\sqrt{2}}\begin{pmatrix}1\\0\\1\\0\end{pmatrix}, \quad \psi_- = \frac{1}{\sqrt{2}}\begin{pmatrix}0\\1\\0\\-1\end{pmatrix}. \tag{89}$$

Projected into the subspace spanned by these two states, Supplementary Equation (79) becomes

$$\frac{E}{\Delta}\sum_{\boldsymbol{r}'}\tilde{M}_1(\boldsymbol{r}-\boldsymbol{r}')\tilde{\psi}(\boldsymbol{r}') = \sum_{\boldsymbol{r}'}\tilde{M}_0(\boldsymbol{r}-\boldsymbol{r}')\tilde{\psi}(\boldsymbol{r}'), \tag{90}$$

$$\tilde{M}_1(\delta\boldsymbol{r}=0) = \eta\tilde{\sigma}_z, \tag{91}$$

$$\tilde{M}_1(\delta\boldsymbol{r}\neq 0) = (\frac{1}{2}J)\tilde{c}_+\tilde{\sigma}_z, \tag{92}$$

$$\tilde{M}_0(\delta\boldsymbol{r}=0) = (1-\eta)\tilde{\sigma}_0, \tag{93}$$

$$\tilde{M}_0(\delta\boldsymbol{r}\neq 0) = (\frac{1}{2}J)\left[-\tilde{c}_+\tilde{\sigma}_0 + \tilde{c}_-(\tilde{\sigma}_x\cos\varphi_r + \tilde{\sigma}_y\sin\varphi_r)\right], \tag{94}$$

where

$$\tilde{M}_i \equiv (\psi_+,\psi_-)^\dagger M_i(\psi_+,\psi_-) \qquad (i=0,1), \tag{95}$$

$\tilde{\psi}$ and $\tilde{\boldsymbol{\sigma}}$ (tildes will be omitted hereafter) are wavefunctions and Pauli matrices under the basis (ψ_+,ψ_-). Fourier transforming Supplementary Equation (90) we obtain

$$H_{\text{eff}}(\boldsymbol{q})\psi(\boldsymbol{q}) = \frac{E}{\Delta}\psi(\boldsymbol{q}), \tag{96}$$

$$H_{\text{eff}}(\boldsymbol{q}) \equiv \left[\sum_{\boldsymbol{r}} e^{-i\boldsymbol{q}\cdot\boldsymbol{r}}\tilde{M}_1(\boldsymbol{r})\right]^{-1}\left[\sum_{\boldsymbol{r}} e^{-i\boldsymbol{q}\cdot\boldsymbol{r}}\tilde{M}_0(\boldsymbol{r})\right]. \tag{97}$$

Or,

$$H_{\text{eff}}(\boldsymbol{q}) = [\eta + d_0(\boldsymbol{q})]^{-1}\left\{[1-\eta-d_0(\boldsymbol{q})]\sigma_z + d_1(\boldsymbol{q})\sigma_x + d_2(\boldsymbol{q})\sigma_y\right\}, \tag{98}$$

$$d_0(\boldsymbol{q}) \equiv \left(\frac{1}{2}J\right)\sum_{\boldsymbol{r}\neq 0} e^{-i\boldsymbol{q}\cdot\boldsymbol{r}}\tilde{c}_+(r), \tag{99}$$

$$d_1(\boldsymbol{q}) \equiv -\left(\frac{i}{2}J\right)\sum_{\boldsymbol{r}\neq 0} e^{-i\boldsymbol{q}\cdot\boldsymbol{r}}\tilde{c}_-(r)\sin\varphi_r, \tag{100}$$

$$d_2(\boldsymbol{q}) \equiv \left(\frac{i}{2}J\right)\sum_{\boldsymbol{r}\neq 0} e^{-i\boldsymbol{q}\cdot\boldsymbol{r}}\tilde{c}_-(r)\cos\varphi_r. \tag{101}$$

Note that d_1 and d_2 are defined with slight differences in the main text in order to make a compact expression.

We can further construct a Majorana basis by using the fact that ψ_+ and ψ_- are particle-hole images of each other:

$$\psi_1^{(M)} = \frac{1}{\sqrt{2}}(\psi_+ + \psi_-) = \frac{1}{2}\begin{pmatrix}1\\1\\1\\-1\end{pmatrix},\ \psi_2^{(M)} = \frac{i}{\sqrt{2}}(\psi_+ - \psi_-) = \frac{i}{2}\begin{pmatrix}1\\-1\\1\\1\end{pmatrix}. \tag{102}$$

Under this Majorana basis, the effective Hamiltonian is given by

$$H_{\text{eff}}^{(M)}(\boldsymbol{q}) = -\left[\eta + d_0(\boldsymbol{q})\right]^{-1}\left\{[1-\eta-d_0(\boldsymbol{q})]\sigma_y + d_2(\boldsymbol{q})\sigma_x - d_1(\boldsymbol{q})\sigma_z\right\}. \tag{103}$$

Clearly, when $\boldsymbol{q}$ is one of the inversion symmetric momenta [ISMs, namely, $(0,0)$, $(0,\pi)$, $(\pi,0)$, or (π,π)], $e^{-i\boldsymbol{q}\cdot\boldsymbol{r}} = e^{i\boldsymbol{q}\cdot\boldsymbol{r}}$, therefore $d_1(\boldsymbol{q}) = d_2(\boldsymbol{q}) = 0$; the Pfaffian function is given by

$$P(\boldsymbol{q}\in \text{ISMs}) = 1 - \frac{1}{\eta + d_0(\boldsymbol{q})} \tag{104}$$

$$\simeq d_0(\boldsymbol{q}) \qquad (\eta \simeq 1). \tag{105}$$

Now return to the effective Hamiltonian of Supplementary Equation (98). If spin-orbit coupling is absent, then $\forall \boldsymbol{q}: \ d_1(\boldsymbol{q}) = d_2(\boldsymbol{q}) = 0$. This immediately implies that if there is any sign change in $P(\boldsymbol{q})$ among the ISMs, the system is gapless with line nodes. Rashba spin-orbit coupling potentially lifts these degeneracies. The sign of the Pfaffian formula (105), multiplied over both C_4 symmetric momenta is equal to the parity of the Chern number. We have evaluated the expression numerically, with the result given in the main text.

In addition we have evaluated the Chern numbers through numerical integrations by using Hamiltonian of Supplementary Equation (98) straightforwardly. Some representative results are shown in Supplementary Figure 1. We confirm the existence of high Chern numbers associated with this effective Hamiltonian, which complements the phase diagram in terms of the parity of the Chern numbers presented in Figure 3 of the main text.

Supplementary Note 3. Two-dimensional Shiba lattice with helical magnetic order

In this section we focus on a different type of magnetic order: we assume a two-dimensional helical pattern for the magnetic moments. For simplicity we neglect the detailed spatial structure of the Shiba states (which is crucial in the previous section) and investigate the following tight-binding Hamiltonian

$$H = \sum_n \left\{ c_n^\dagger (\boldsymbol{B}_n \cdot \boldsymbol{\sigma} - \mu) c_n + [\Delta_n c_n^T (\mathrm{i}\sigma_y) c_n + h.c.] + \sum_{\delta=\pm 1_x, \pm 1_y} t_{n\delta}\, c_n^\dagger c_{n+\delta} \right\}, \tag{106}$$

where n is a 2D index for the tight-binding sites, $c_n = (c_{n\uparrow}, c_{n\downarrow})^T$ is the vector of electron annihilation operators at site n, $\boldsymbol{B}_n$ stands for the local magnetic moment coupled to the superconductor electron spin, Δ_n stands for the local pairing potential, and $t_{n\delta}$ stands for spin-independent nearest-neighbor hoppings.

By changing to a rotating basis $g_n = U_n^\dagger c_n$ with U_n defined by

$$U_n^\dagger (\boldsymbol{B}_n \cdot \boldsymbol{\sigma}) U_n = B_n \sigma_z, \quad U_n^\dagger U_n = \sigma_0, \quad \mathrm{Det}\, U_n = 1, \tag{107}$$

the above Hamiltonian becomes

$$\tilde{H} = \sum_n \left\{ g_n^\dagger (B_n \sigma_z - \mu) g_n + [\Delta_n g_n^T (\mathrm{i}\sigma_y) g_n + h.c.] + \sum_{\delta=\pm 1_x, \pm 1_y} t_{n\delta}\, g_n^\dagger \Omega_{n\delta} g_{n+\delta} \right\}, \tag{108}$$

where

$$\Omega_{n\delta} = U_n^\dagger U_{n+\delta}, \quad \mathrm{Det}\, \Omega_{n\delta} = 1. \tag{109}$$

To further simplify the problem, we set $B_n = B$, $\Delta_n = \Delta_n^* = \Delta$, $t_{n1_x} = t_{n1_x}^* = t_x$, $t_{n1_y} = t_{n1_y}^* = t_y$ for all n.

We also assume a certain periodical pattern for the magnetic moments $\hat{B}_n$. One simplest, nevertheless quite general, choice is to let

$$\Omega_{(n_x,0),\delta=1_x} = \Omega_x = \exp[i(\rho_x/2)\hat{r}_x \cdot \boldsymbol{\sigma}], \tag{110}$$

$$\Omega_{(n_x,n_y),\delta=1_y} = \Omega_y = \exp[i(\rho_y/2)\hat{r}_y \cdot \boldsymbol{\sigma}], \tag{111}$$

for all n_x and n_y. Here $\rho_{x,y}$ stand for rotation angles between neighboring sites and $\hat{r}_{x,y}$ stand for rotation axes. The rest of the Ω-matrices are hereby determined by a closed-path formula:

$$\Omega_{(n_x,n_y),\delta=1_x} = \Omega_x^{(n_y)} = \Omega_y^{-n_y}\Omega_x\Omega_y^{n_y} = \exp[i(\rho_x/2)\Omega_y^{-n_y}(\hat{r}_x\cdot\boldsymbol{\sigma})\Omega_y^{n_y}]. \tag{112}$$

If Ω_x and Ω_y do not commute and $\rho_y = 2\pi p/q$ [which implies $\Omega_y^q = (-\mathbb{1})^p$] with p and q relatively prime integers, then $\Omega_x^{(n_y)} = \Omega_x^{(n_y \mod q)}$; each unit cell consists of q sites. The Bloch Hamiltonian in the Nambu basis is given by

$$H_k = \boldsymbol{g}_k^\dagger \begin{pmatrix} h(B,\boldsymbol{k}) & \Delta \\ \Delta & -h(-B,\boldsymbol{k}) \end{pmatrix} \boldsymbol{g}_k, \tag{113}$$

$$\boldsymbol{g}_k^\dagger = (g_{0,k\uparrow}^\dagger, g_{0,k\downarrow}^\dagger, \dots, g_{q-1,k\uparrow}^\dagger, g_{q-1,k\downarrow}^\dagger, g_{0,-k\downarrow}, -g_{0,-k\uparrow}, \dots, g_{q-1,-k\downarrow}, -g_{q-1,-k\uparrow}), \tag{114}$$

$$h(B,\boldsymbol{k}) = \begin{pmatrix} h_0(B,k_x) & \tau(k_y) & 0 & \tau(k_y)^\dagger \\ \tau(k_y)^\dagger & h_1(B,k_x) & \tau(k_y) & 0 \\ 0 & \ddots & \ddots & \ddots \\ \tau(k_y) & 0 & \tau(k_y)^\dagger & h_{q-1}(B,k_x) \end{pmatrix}, \tag{115}$$

$$h_{n_y}(B,k_x) = B\sigma_z - \mu + t_x(\Omega_x^{(n_y)}e^{ik_x} + h.c.), \tag{116}$$

$$\tau(k_y) = t_y\Omega_y e^{ik_y/q}. \tag{117}$$

The above model is similar, but in general not equivalent, to a model with constant $\boldsymbol{B}_n$ and spin-orbit coupling, such as the following one:

$$h_{SO}(B,\boldsymbol{k}) = \frac{\hbar^2k^2}{2m} - \mu + B\sigma_z + k_x(\boldsymbol{\alpha}_x\cdot\boldsymbol{\sigma}) + k_y(\boldsymbol{\alpha}_y\cdot\boldsymbol{\sigma}), \tag{118}$$

where $\boldsymbol{\alpha}_x$ and $\boldsymbol{\alpha}_y$ are real-valued vectors. To see this, we write the Hamiltonian of Supplementary Equation (118) on a lattice. The hopping terms have the form

$$\langle n|h_{SO}|n+1_{l=x,y}\rangle = t + \frac{1}{2i}\boldsymbol{\alpha}_l\cdot\boldsymbol{\sigma}. \tag{119}$$

If we define

$$t_{l=x,y} = \sqrt{t^2+|\boldsymbol{\alpha}_l/2|^2}, \quad \Omega_{l=x,y} = (t+\frac{1}{2i}\boldsymbol{\alpha}_l\cdot\boldsymbol{\sigma})/t_l, \tag{120}$$

where $\Omega_{l=x,y}$ are unitary and $\mathrm{Det}\,\Omega_l = 1$, this Hamiltonian formally resembles Hamiltonian of Supplementary Equation (108) except that, importantly, the Ω's such defined do not necessarily satisfy a closed-path equation as Supplementary Equation (112).

A. Commuting helix rotations

Obviously, Ω_x and Ω_y commute when $\hat{r}_x = \hat{r}_y = \hat{r}$. In this case

$$h(B, \boldsymbol{k}) = B\sigma_z - \mu + t_x(\Omega_x e^{ik_x} + h.c.) + t_y(\Omega_y e^{ik_y} + h.c.). \tag{121}$$

Obviously we can rotate the basis again such that $\hat{r} = (\sin\theta, 0, \cos\theta)$. Then

$$h(B, \boldsymbol{k}) = \begin{pmatrix} B - v - m & -u \\ -u & -(B - v) - m \end{pmatrix}, \tag{122}$$

$$m = \mu - 2t_x \cos\frac{\rho_x}{2}\cos k_x - 2t_y\cos\frac{\rho_y}{2}\cos k_y, \tag{123}$$

$$u = (2t_x \sin\frac{\rho_x}{2}\sin k_x + 2t_y \sin\frac{\rho_y}{2}\sin k_y)\sin\theta, \tag{124}$$

$$v = (2t_x \sin\frac{\rho_x}{2}\sin k_x + 2t_y \sin\frac{\rho_y}{2}\sin k_y)\cos\theta. \tag{125}$$

Let us assume $\theta = \pi/2$, the spectrum of the Nambu Hamiltonian is given by

$$E = \pm\sqrt{B^2 + \Delta^2 + m^2 + u^2 \pm 2\sqrt{B^2\Delta^2 + B^2m^2 + m^2u^2}}. \tag{126}$$

The conditions for $E = 0$ are

$$2t_x \sin\frac{\rho_x}{2}\sin k_x + 2t_y \sin\frac{\rho_y}{2}\sin k_y = 0, \tag{127}$$

$$\mu = 2t_x\cos\frac{\rho_x}{2}\cos k_x + 2t_y\cos\frac{\rho_y}{2}\cos k_y \pm \sqrt{B^2 - \Delta^2}. \tag{128}$$

Considering $B \gg \max(\Delta, t)$ and $\mu > 0$, the spectrum is clearly gapped when $|\mu - \sqrt{B^2 - \Delta^2}| > 2t_x|\cos\frac{\rho_x}{2}| + 2t_y|\cos\frac{\rho_y}{2}|$; otherwise the spectrum is gapless with two Fermi points $\pm(k_{x0}, k_{y0})$ [see e.g. Supplementary Figure 2(a)] that merge when the equal sign is taken.

B. Non-commuting helix rotations

Now we consider the case when Ω_x and Ω_y do not commute. The most important feature of the band structure is that the subbands created by the multiple sites of a unit cell are *not* gapped. To see this, let us look at the Hamiltonian of Supplementary Equation (113) at $k_x = 0$ or π, where $e^{ik_x} = \pm 1$, and

$$\Omega_x^{(m)} e^{ik_x} + h.c. = \pm(\Omega_x^{(m)} + h.c.) = \pm 2\cos(\rho_x/2). \tag{129}$$

This implies that $h_m(B,k_x)$ has no dependence on m at $k_x=0$ or π; the unit cell is essentially of a single site. Explicitly, the Hamiltonian becomes

$$H_{k_x=0,\pi}(k_y) = \sum_{m=0}^{q-1} \tilde{\boldsymbol{g}}_{mk_y}^\dagger \begin{pmatrix} \tilde{h}_m(B,k_y) & \Delta \\ \Delta & -\tilde{h}_m(-B,k_y) \end{pmatrix} \tilde{\boldsymbol{g}}_{mk_y}, \tag{130}$$

$$\tilde{\boldsymbol{g}}_{mk}^\dagger = (\tilde{g}_{m,k\uparrow}^\dagger, \tilde{g}_{m,k\downarrow}^\dagger, \tilde{g}_{m,-k\downarrow}, -\tilde{g}_{m,-k\uparrow}), \quad \tilde{g}_{m,k\sigma} = \sum_{m'=0}^{q-1} \frac{1}{\sqrt{q}} e^{-i\frac{2\pi m m'}{q}} g_{m',k\sigma}, \tag{131}$$

$$\tilde{h}_m(B,k_y) = B\sigma_z - \mu \pm 2t_x \cos(\rho_x/2) + \tau(2\pi m + k_y) + \tau(2\pi m + k_y)^\dagger. \tag{132}$$

The crossings of the subbands do not necessarily occur at $k_y = 0$ or π unless there is an inversion symmetry. One example is that if $\Omega_y = \exp[i(\rho_y/2)(\sigma_x \cos\varphi_y + \sigma_y \sin\varphi_y)]$, then $\sigma_z\Omega_y\sigma_z = \Omega_y^\dagger$, and $\sigma_z \tilde{h}_m(B,k_y)\sigma_z = \tilde{h}_{q-m}(B,-k_y)$. In other words, if the rotation axis $\hat{r}_y$ lies in the x-y plane, then the crossings of the subbands occur at $k_y = 0$ or π.

As a special case, we set $\rho_y = \pi$, $\hat{r}_y = (1,0,0)$, $\hat{r}_x = (\cos\varphi_x, \sin\varphi_x, 0)$. In this case, each unit cell consists of two sites, $\Omega_y = i\sigma_x$, $\Omega_y^{-1}\Omega_x\Omega_y = \Omega_x^T$, and (we also set $t_x = t_y = t$)

$$h(B,\boldsymbol{k}) = \begin{pmatrix} B\sigma_z - \mu + t(\Omega_x e^{ik_x} + h.c.) & -2t\sigma_x \sin k_y/2 \\ -2t\sigma_x \sin k_y/2 & B\sigma_z - \mu + t(\Omega_x^T e^{ik_x} + h.c.) \end{pmatrix}. \tag{133}$$

The spectrum of this Hamiltonian can be solved to be

$$E = \pm\sqrt{B^2 + \Delta^2 + m_k^2 + d_{k\pm}^2 \pm 2\sqrt{B^2\Delta^2 + B^2 m_k^2 + m_k^2 d_{k\pm}^2}}, \tag{134}$$

$$m_k = \mu - 2t\cos\frac{\rho_x}{2}\cos k_x, \tag{135}$$

$$d_{k\pm} = 2t(\sin\frac{\rho_x}{2}\sin k_x \pm \sin\frac{k_y}{2}). \tag{136}$$

The conditions for $E = 0$ are

$$\sin\frac{\rho_x}{2}\sin k_x \pm \sin\frac{k_y}{2} = 0, \tag{137}$$

$$\mu = 2t\cos\frac{\rho_x}{2}\cos k_x \pm \sqrt{B^2 - \Delta^2}. \tag{138}$$

Considering $B \gg \max(\Delta, t)$ and $\mu > 0$, the spectrum is gapped only when $\mu > |2t\cos\frac{\rho_x}{2}| + \sqrt{B^2-\Delta^2}$ or $\mu < -|2t\cos\frac{\rho_x}{2}| + \sqrt{B^2-\Delta^2}$. When $|\mu - \sqrt{B^2-\Delta^2}| < |2t\cos\frac{\rho_x}{2}|$, there are always four Fermi points [see e.g. Supplementary Figure 2(b)].

With more general helical patterns, full superconducting gaps can be opened with nontrivial topological properties described by Chern numbers [2]. The Chern number, as well as the spectrum

for an open-boundary sample, can be computed numerically. Unless otherwise stated, we set $B = 5$, $t_x = t_y = 1$, $\hat{r}_y = (1, 0, 0)$ and $\hat{r}_x = (0, -1, 0)$ in our computations. We shown in Supplementary Figure 3 several examples of the phase diagrams in terms of the Chern numbers with different helical patterns. In Supplementary Figure 4 we show in addition an example of bulk and edge spectrum for a fixed combination of μ and Δ.

Supplementary References

[1] Pientka, F., Glazman, L. I. & von Oppen, F. Topological superconducting phase in helical Shiba chains. *Physical Review B* **88**, 155420 (2013).

[2] Nakosai, S., Tanaka, Y. & Nagaosa, N. Two-dimensional p-wave superconducting states with magnetic moments on a conventional s-wave superconductor. *Physical Review B* **88**, 180503 (2013).
